# Natural variability and individuality of walking behavior in *Drosophila*

**DOI:** 10.1242/jeb.247878

**Published:** 2024-11-21

**Authors:** Vincent Godesberg, Till Bockemühl, Ansgar Büschges

**Affiliations:** Department of Animal Physiology, Institute of Zoology, University of Cologne, 50674 Cologne, Germany

**Keywords:** Variability, Motor control, Legged locomotion, Insect, Inter-leg coordination

## Abstract

Insects use walking behavior in a large number of contexts, such as exploration, foraging, escape and pursuit, or migration. A lot is known about how nervous systems produce this behavior in general and also how certain parameters vary with regard to walking direction or speed, for instance. An aspect that has not received much attention is whether and how walking behavior varies across individuals of a particular species. To address this, we created a large corpus of kinematic walking data of many individuals of the fruit fly *Drosophila*. We only selected instances of straight walking in a narrow range of walking speeds to minimize the influence of high-level parameters, such as turning and walking speed, aiming to uncover more subtle aspects of variability. Using high-speed videography and automated annotation, we captured the positions of the six leg tips for thousands of steps and used principal components analysis to characterize the postural space individuals used during walking. Our analysis shows that the largest part of walking kinematics can be described by five principal components (PCs). Separation of these five PCs into a 2D and a 3D subspace divided the description of walking behavior into invariant features shared across individuals and features that relate to the specifics of individuals; the latter features can be regarded as idiosyncrasies. We also demonstrate that this approach can detect the effects of experimental interventions in an unbiased manner and that general aspects of individuality, such as the individual walking posture, can be described.

## Teaser

**Summary:** The kinematics of walking behavior in *Drosophila* can be decomposed into general aspects of motor output and idiosyncrasies of individual flies.

## INTRODUCTION

Legged locomotion, commonly subsumed under the term walking, is found in most terrestrial animals. Walking is used in a diverse set of behavioral contexts, such as exploration and foraging, escape, pursuit, mating and migration, making it a central component of an animal's behavioral repertoire. The diversity of these contexts requires walking to be highly adaptable and flexible. Consequently, the task- and situation-specific neuronal control of walking behavior is important for its proper execution.

The neuronal control and kinematics of walking have been extensively studied in a large variety of arthropods (Nirody, 2021, 2023), particularly in insects, from small insects, such as fruit flies (*Drosophila melanogaster*: DeAngelis et al., 2019; Mendes et al., 2013, 2014; Strauß and Heisenberg, 1990; Wosnitza et al., 2012) or desert ants (*Cataglyphis*: Pfeffer et al., 2019; Wahl et al., 2015; Zollikofer, 1994), to large ones, such as cockroaches (*Periplaneta americana*: Couzin-Fuchs et al., 2015; Delcomyn, 1971, 1989), locusts (*Schistocerca gregaria*: Burns, 1973; Niven et al., 2010; Pearson and Franklin, 1984) or stick insects (*Carausius morosus*: Cruse, 1976; Dallmann et al., 2016; Dürr and Ebeling, 2005; Gruhn et al., 2009). There is a large body of knowledge about these groups, ranging from the sensorimotor control of individual legs, to how inter-leg coordination of the six legs is achieved, to high-level descending and central neuronal control (Bidaye et al., 2018; Cruse, 1990; Dürr et al., 2004). A number of common features governing the neuronal and kinematic aspects of insect walking have been identified. Walking speed, as a major aspect, seems to be generally controlled by changes in stance duration, while stance amplitude and swing duration are largely kept constant (DeAngelis et al., 2019; Wosnitza et al., 2012). These changes in stance duration are accompanied by systematic changes in inter-leg coordination. Unlike larger vertebrates, however, which use distinct gaits in a speed-dependent manner (Diedrich and Warren, 1995; Hoyt and Taylor, 1981), insects exhibit a continuum of inter-leg coordination patterns (Szczecinski et al., 2018; Wosnitza et al., 2012). These systematic effects and other invariant features are generally present in walking insects as an evolved phenotypic trait; however, the neuronal control and kinematics of walking must have exhibited hereditary interindividual and inter-species variability during evolution, thereby adapting to co-evolving traits such as body morphology or ecological demands. Indeed, a previous study on the evolution of walking behavior in a large set of drosophilids showed that there exist systematic differences in walking behavior across species and strains (York et al., 2022). These differences evolve and diverge rapidly in closely related species, but re-converge to shared features in more distantly related ones (York et al., 2022). However, while that previous study establishes an evolutionary approach to investigate the variability of walking in insects, it was mainly based on the characteristics of the velocities at which different species walk and did not focus more specifically on detailed leg kinematics. Thus, how variable walking is within and between individuals of a given species or strain on a level closer to the actual motor output is still largely unexplored.

Walking behavior differs in multiple parameters between individual flies, such as average posture, preferred coordination pattern, walking speed or degree of intraindividual variability, as anecdotal observations indicated. However, a quantitative description of these differences is still lacking. To explore this aspect in greater detail, in the present study we wanted to explicitly acknowledge variability as an important aspect of walking behavior on the interindividual and intraindividual level and to find an unbiased and more comprehensive way of characterizing and interpreting the observed variability. Behavioral variability on the individual level is a topic that has started to receive more attention in recent years. For instance, two studies on handedness in *Drosophila* show that individual flies can have left/right preferences in a Y-maze decision paradigm and that these preferences are partly hereditary (Ayroles et al., 2015; Buchanan et al., 2015). A study on several *Drosophila* wild-type strains established systematic differences in odor preference that can be traced back to phenotypic differences related to these strains (Ruebenbauer et al., 2008). Further studies in *Drosophila* showed that grooming behavior shows variability between different wild-type lab strains, but also between isogenic individuals of the same strain (Mueller et al., 2022), and that the kinematics of trajectories during object orientation are highly individualized and also that neuronal asymmetries drive this behavioral individuality (Linneweber et al., 2020).

Here, we investigated the natural intraindividual and interindividual variability of low-level kinematic parameters of walking in the fruit fly *Drosophila melanogaster*. To control for and exclude known influences of walking speed and curve walking on kinematics, we initially focused on straight walking at intermediate speeds in a large set of male flies, each of which spontaneously produced a large corpus of walking behavior in an unrestrained free-walking paradigm. Using high-speed video recording and automated annotation based on deep learning methods (DeepLabCut, DLC; Mathis et al., 2018), we extracted the positions of two body markers, as well as the tarsal tips from all video frames in these straight sequences, automatically determined positions and times of lift-off and touch-down events of the legs, and calculated walking speed and inter-leg coordination for all step cycles. Across individuals, the dataset we created in this way contained more than 36,000 steps for each leg. We used principal components analysis (PCA) to find a compact description of this large dataset and systematically explored correlations and the variability between leg kinematics on an individual basis as well as across individuals. In the field of motor control, biomechanics and kinematics, PCA has been used to extract motor synergies in the context of cortical control of hand movements in monkeys (Mollazadeh et al., 2014) and joint-angle correlations of targeted catching movements in humans (Bockemühl et al., 2010), to detect altered kinematic profiles in stroke patients (Milovanović and Popović, 2012), to characterize recovery of locomotor function after spinal injury in mice (Takeoka et al., 2014; Takeoka and Arber, 2019) and to evaluate the complexity of wing kinematics in bats (Riskin et al., 2008). In *Drosophila* locomotion, PCA has been applied, for instance, to evaluate the effect of neurotoxins on a large number of kinematic parameters during walking (Cabrita et al., 2022). Furthermore, an approach mathematically related to PCA has been used to extract a set of wing movement patterns that allow for flight control in *Drosophila* (Chakraborty et al., 2015).

Here, PCA revealed that most of the kinematic variability in our dataset (approximately 80%) is contained in the first five PCs. We show that two subsets of these five PCs describe interindividually applicable dynamics of inter-leg coordination, on the one hand, and individual characteristics of walking behavior, what might be called idiosyncrasies, on the other. The first subset contains two PCs which mainly capture inter-leg coordination-specific aspects of walking – how tripod-like a particular movement pattern is or the general repetitive sequence of alternating swing and stance movements of individual legs. In contrast, the contribution of a second subset of three PCs relates to how individuals differ from each other in the way they walk; data for different individuals occupy different regions within this PC subspace, highlighting interindividual differences. Simultaneously, the same flies are indistinguishable in the subspace related to inter-leg coordination, supporting the notion that these two PCs describe universal aspects of intraindividual variability. The importance and applicability of these two subsets of PCs is further substantiated by (1) relating them to a quantitative measure of tripod coordination strength (TCS), (2) showing that individual-specific contributions to walking remain constant with regard to walking speed, (3) a use case for characterizing changes in walking behavior induced by optogenetic inhibition of sensory structures in the legs and, finally, (4) deriving a 3D measure for postural adaptations. Our results suggest that the variability observed in walking flies is systematic and that PCA is a suitable approach for the quantification of and decomposition into idiosyncrasies, inter-leg coordination patterns, and effects of experimental interventions.

## MATERIALS AND METHODS

### Fly strains and husbandry

Male flies of the wild-type strain Berlin-K [Bloomington Drosophila Stock Center (BDSC), #8522] were used for those experiments which formed the basis for PCA (see below). Inhibition experiments (see below) were performed with F1 flies resulting from crosses between *iav*-Gal4 (O'Dell and Burnet, 1988) (BDSC #52273) and UAS-GtACR1 (Govorunova et al., 2015; Mohammad et al., 2017) (BDSC #92983). The use of both the wild-type strain Berlin-K and the *iav*-Gal4 line owes to the fact that we wanted to have a direct connection with the data and results from a previous study (Chockley et al., 2022).

Wild-type flies were raised on 12 h/12 h light/dark cycle, while transgenic flies were raised in the dark to prevent premature activation of GtACR1 (*Guillardia theta* anion channelrhodopsin 1; see below) channels and potential adaptation prior to experiments. All flies were kept at 25°C and approximately 60% humidity on a standard food medium (Backhaus et al., 1984). To improve the function of GtACR1, transgenic flies had 60 µg of all-trans-retinal in their food for at least 3 days prior to the experiments. All animals used in this study were 5 days old. To increase walking activity, flies were isolated and starved for approximately 24 h before experiments, but had access to moist tissue paper during this period.

### Experimental setup

**Fig. 1.**
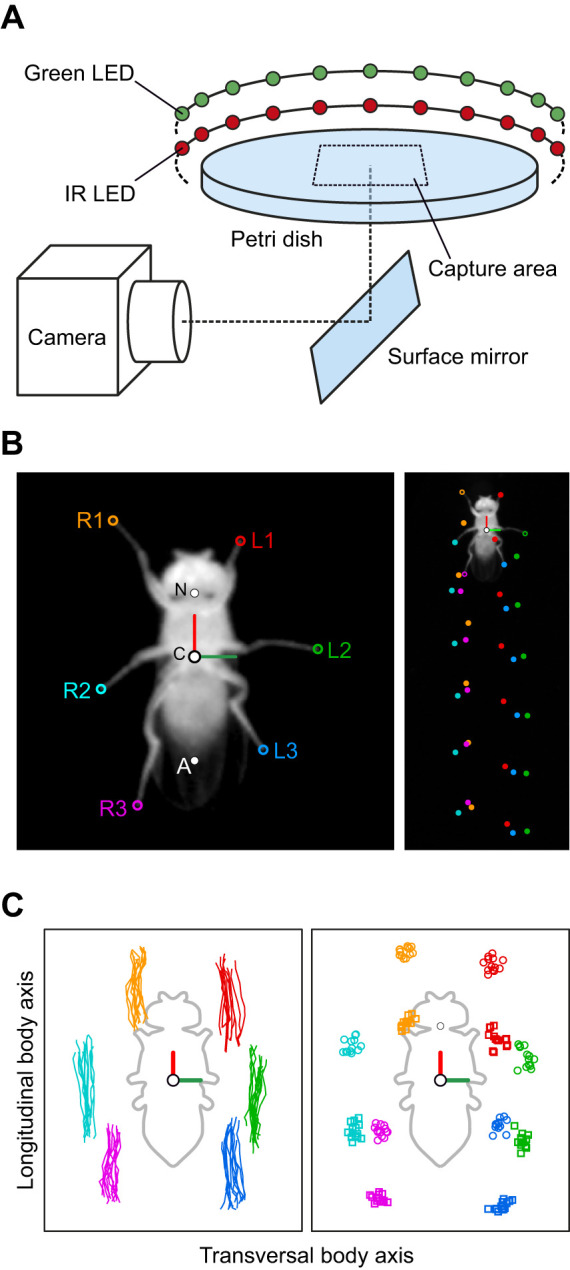
**Experimental setup and data acquisition.** (A) Schematic diagram of the experimental setup. The watch glass covering the Petri dish is not depicted. (B) Examples of the body-centered bottom view (left) and the camera view (right) with annotated leg tips (right legs R1–R3, and left legs L1–L3), abdomen (A), center of the body (C) and neck (N). (C) Examples of stance trajectories (left) and tarsus extreme positions (right) of one walking bout after detection of lift-off and touch-down positions. Circles: anterior extreme positions (AEPs), squares: posterior extreme positions (PEPs). The fly silhouette (gray outline) is shown for reference.

The experimental setup described here is largely identical to the one used in a previous study (Chockley et al., 2022). However, for clarity, we describe it in detail here again. The recording arena (Fig. 1A) consisted of an inverted glass Petri dish (diameter: 60 mm) as the walking substrate and a watch glass (diameter: 100 mm) as the lid. This arrangement formed a closed chamber with a curved dome tapered towards the edge of the Petri dish, similar to an inverted FlyBowl (Simon and Dickinson, 2010). The inner side of the watch glass was coated with SigmaCote (SL2, Sigma-Aldrich, St Louis, MO, USA). This resulted in a hydrophobic surface on which flies found less grip; walking on the ceiling was thus reduced. The Petri dish and the watch glass were placed in a custom-made plastic holder with a cutout that allowed for video recordings from below (holder not shown in Fig. 1A). To record flies walking on the glass substrate, we used a camera (model VC-2MC-M340, Vieworks, Anyang, Republic of South Korea) equipped with an object-space telecentric lens (focal length 55 mm, model Computar TEC-55, CBC America, Cary, NC, USA). The telecentric lens provided an orthographic projection, reducing image position-dependent changes in apparent fly posture. The camera was located on the side of the setup and its view was directed at the experimental chamber from below via a surface mirror tilted at an angle of 45 deg. To increase video resolution, the camera view was focused on a square area in the center of the walking substrate (30 mm side length, resolution 1000×1000 pixels, 33.3 pixels mm^−1^). The typical body length (BL, defined as the distance between the anterior end of the head and the tip of the abdomen) of a fly of approximately 2 mm corresponded therefore to 65–70 pixels; this was sufficient to image all body parts annotated during analysis with sufficient accuracy, particularly the tarsal tips (Fig. 1B, left). The scene was illuminated with 60 infrared (IR) LEDs (wavelength 890 nm, opening angle 20 deg) arranged in a concentric ring around the chamber. Light from these LEDs was emitted mainly parallel to the arena surface. Thus, only the fly reflected any appreciable amount of light, resulting in a strong contrast between it and the background (see Fig. 1B). As ambient light during experiments was generally low, flies walked largely in the dark. However, contrast was further enhanced by adding a visible-light filter (upper cut-off frequency 790 nm) to the camera's lens, eliminating any remaining ambient light from the room or optogenetic inhibition (see below). Video data were acquired at 200 Hz and a shutter time of 400 µs; this low shutter time prevented motion blur and ensured detectability of the leg tips even during very fast movements. Acquisition of individual video frames during this shutter time and IR illumination were synchronized with a pulse generator. For inhibition experiments (see below), we added a second LED ring around the chamber. This ring consisted of 60 green LEDs (wavelength 525 nm) whose light was directed at the recording chamber. These LEDs could be switched on and off programmatically during an ongoing experiment via a multi-function I/O device (USB-6001, National Instruments Corporation, Austin, TX, USA). The experimental setup was custom designed and built (Electronics workshop, Department of Animal Physiology, University of Cologne).

### Behavioral experiments

Prior to an experiment, single flies were aspirated into a tube and then transferred into the arena; no CO_2_ was used for this step. Flies were then allowed to acclimate to the setup for 5 min prior to the start of video acquisition. The total duration of an experiment was up to 3 h. During this time, flies walked spontaneously in the chamber and frequently crossed the capture area. For the complete duration of an experiment, video data were acquired continuously and the last 1000 frames (equivalent to 5 s) were stored in a ring buffer. Custom-written software functions evaluated the recorded frames in real-time and determined whether the fly was present in a particular video frame and whether it had produced a continuous walking track with a minimum length of 3 BL and a minimum walking speed of 2 BL s^−1^. Once the fly had produced such a track and then stopped or left the capture area, the contents of the frame buffer were committed to storage as a valid trial. After this, acquisition automatically started anew. Note that at this time no additional selection criteria (such as curvature or walking speed, for instance) were applied to determine the validity of a trial. The set of videos acquired in this way merely served as a large and relatively unconstrained initial set of walking behavior (see next section on further criteria for data inclusion).

The inhibition experiments with *iav*-Gal4>UAS-GtACR1 used for evaluation of the descriptive power of PCA (see below) were performed in the same setup. Technically, these experiments are a replication of those conducted in one of our previous studies (see fig. 3 of Chockley et al., 2022). In this previous study, we confirmed that the wild-type strain Berlin-K used in the present study did not show strong changes in leg kinematics simply as a result of exposure to green light (see fig. S2 in Chockley et al., 2022). We therefore restricted our inhibition experiments in the present study to *iav*-Gal4>UAS-GtACR1 and extended the experiments as follows. Flies in the inhibition experiments either walked in the dark (wild-type control condition) or walked under green-light illumination (inhibition condition). Trials in the dark and in the light were alternated; once the fly had produced a trial in the dark, the green-light illumination was switched on and the system was primed to record the next trial. If the fly did not produce a valid trial within the first 60 s of the green-light condition, the light was switched off and data acquisition was suspended for 30 s to prevent adaptation to the inhibition and potentially harmful effects of prolonged exposure to the green light. After this cooldown period, the green light was switched on again, and data acquisition was resumed. Once a trial had eventually been recorded in the inhibition condition, the light was switched off again and the process was repeated. Data from these inhibition experiments were sorted into control trials (recorded in the dark) and inhibition trials (recorded during green-light illumination). Video acquisition, online data evaluation during experiments and high-level hardware control were implemented with custom-written software in MATLAB (2018b, The MathWorks, Natick, MA, USA).

### Processing of video data

Because curve walking has a strong influence on leg kinematics in insects (Dürr and Ebeling, 2005; Gruhn et al., 2008; Jander, 1985), we initially restricted our analyses to straight walking. Instances of this in the complete set of walking trials (see previous section) were detected with custom-written algorithms, whose parameters were determined empirically (see Supplementary Materials and Methods). Only segments of video data associated with straight walking were extracted from valid trials. In each video frame of these segments, eight different body parts (the tarsal tips of all legs, the neck and the posterior tip of the abdomen) were detected with DLC (Mathis et al., 2018) (Fig. 1B). To improve the robustness of DLC, we first detected the general position of the fly within a video frame using a threshold operation, conversion to a binary image and, finally, calculation of the centroid of the largest contiguous region of white pixels (which corresponded to the fly). Using this position, we then cropped the fly from the video. With these cropped and fly-centered views, we used three different instances of DLC in a two-step analysis. The first step was to detect the neck and abdomen of the fly. These positions were used to define a fly-centric coordinate system (Fig. 1C); all data presented in this study (apart from the identification of swing and stance phases) are based on these body-centric coordinates. The positions of all body parts were then normalized to each fly's body length to allow for body size-independent comparisons between flies. We also used the neck and abdomen positions to rotate the cropped views and align the fly's longitudinal axis vertically. These cropped and rotated data were then used in the second DLC analysis, in which the tarsal tips of each body side were detected by two independently trained instances of DLC, one for the left legs and one for the right legs. In general, DLC performance was very good; to ensure the highest accuracy, however, we also visually inspected all automatically generated annotations for errors and corrected these manually, where necessary.

Swing and stance phases of all legs were determined automatically based on the respective speeds at which tarsal tips moved in an arena-centric coordinate system: whenever a tarsus is stationary in this coordinate system, i.e. it co-moves with the ground, we assumed the leg to be in stance phase (Fig. 1B, right). Conversely, movements of more than 1.5 pixels per frame were empirically defined as swing phase activity (see Supplementary Materials and Methods). A transition between stance and swing phase was defined as a lift-off event. The last position of the tarsal tip on the ground before lift-off was defined as the posterior extreme position (PEP) for that step (Fig. 1C, right). Conversely, a transition between swing and stance phase was defined as a touch-down event; the first tarsal position with ground contact associated with this event was identified as the anterior extreme position (AEP) (Fig. 1C, right). The time of onset of a particular step was defined as its lift-off, and a complete step of a leg was defined as its movement between two consecutive lift-off events, i.e. a swing phase followed by a stance phase. In contrast to steps of individual legs, step cycles (SCs) were defined as follows: the start and end of a SC were determined by the respective step of the right middle leg, which was selected arbitrarily for this purpose. All six tarsal tip positions for this interval comprised the data of one SC. As the stepping period of the six legs in straight walking flies is almost identical and constant for small time windows, each leg completes its own cycle during a SC, although they all start and end at individual positions and phases. In other words, all six leg tip positions at the beginning and the end of a SC are usually highly similar, not just for the reference leg.

The walking speed associated with a step or a SC was defined as the average walking speed of the animal between onset and offset and was used to allow for the selection of steps and SCs within a certain range of walking speeds for analysis. The main body of data used in the present study was based on steps and SCs whose associated walking speed was between 5 and 7 BL s^−1^ (see Fig. S1 for all speed ranges of all individuals). Initially, we restricted the range of walking speeds in this way to facilitate comparability between individuals. Furthermore, previous studies have shown that walking speed has a strong and systematic influence on many of the kinematic parameters investigated here (Mendes et al., 2013; Strauß and Heisenberg, 1990; Wosnitza et al., 2012); this general influence might have an unwanted effect on the initial analysis if individuals walk at different preferred speeds. However, for a later analysis, we expanded the range of walking speeds to lower and higher speeds (see below).

### PCA

PCA is a tool for dimensionality reduction and can be used to find linear correlations of multiple parameters in high-dimensional datasets. Mathematically, calculating PCA is identical to finding the eigenvectors and eigenvalues of a dataset's covariance matrix (Manly and Alberto, 2019). The principal components (PCs) form a new coordinate system that is aligned with the directions of highest variability in the dataset. Importantly, PCs are ordered according to their relevance, i.e. the relative amount of variance they describe.

To evaluate whether a given PC describes a fraction of variability that is meaningful, i.e. larger than what would be expected by chance, a reference data matrix can be constructed that contains the original data but in which the columns (corresponding to the individual variables) are randomly and independently permuted. This removes any correlations between rows (individual observations). Repeating the PCA for this randomized dataset will give eigenvalues for each PC that can serve as reference levels whose values need to be exceeded for a PC of the original data to be identified as meaningful.

We applied PCA to the positions of all six leg tips during SCs for 88 individual flies; 30 SCs between 5 and 7 BL s^−1^ for each of these 88 flies were used as the basis for PCA. Resampling and interpolation were used to acquire exactly 100 postures for each SC in the analysis, resulting in matrices of 3000×12 data points per fly, with each row representing *x*- and *y*-coordinates of the six leg tips and a total of 30 (SCs) times 100 (normalized number of data points per cycle) rows. The final data matrix for PCA comprising data from all flies contained 2640 SCs, represented by 264,000 data points with 12 parameters each. Prior to the analysis, each column (equivalent to one parameter) was standardized to a mean of 0 and unit variance (equivalent to *z*-scores). PCA was carried out on this standardized matrix in MATLAB 2018b (function *pca.m*). To establish reference levels for meaningful contributions of PCs, we used the same data in a randomized second PCA as outlined above.

Here, PCs describe spatial covariation of the positions of all six tarsal tips and can readily be interpreted as movements of the tarsal positions when multiplied by a non-zero factor. Using this fact, individual PCs were visualized by varying their values systematically from −2 to +2 of their respective standard deviations before transferring the data back into the original parameter space. The resulting positional changes are depicted as arrows to indicate the direction of covariation. In mathematical terms, these arrows correspond to the loadings of each PC. For subsequent data analysis, the original data were transformed either in their entirety (264,000×12 matrix) or on a per-fly basis (3000×12) into the new coordinate system established by the PCs. In the context of PCA, these transformed data are typically also referred to as scores.

### PCs and TCS

The fraction of variability described by a PC for one complete cycle of both tripod groups was compared with the respective coordination pattern. TCS, as used in previous studies (e.g. Ramdya et al., 2017; Wahl et al., 2015; Wosnitza et al., 2012), was used to quantify the synchronicity of the swing activity in the two tripod groups. TCS was calculated as follows: for each tripod group (a set of ipsilateral front and hind legs and the contralateral middle leg), the time at which all three legs were simultaneously in swing phase was divided by the time from the earliest swing onset to the latest swing termination in any of these three legs. Hence, a perfect overlap of all three swing phases resulted in a maximal TCS value of 1 and would correspond to canonical tripod coordination. The minimal TCS value of 0 was assigned in cases of no overlap between swing phases in a tripod group. The TCS values of the tripod groups were averaged and the fractions of variability described by each PC for all positions during the movements of these two tripod groups were calculated. This was achieved by transferring all leg tip positions in this respective time frame into the PC space and measuring the fraction of variability described by each PC.

### Analysis of different walking speeds and time of recording

To test how the results of our PCA analysis change for different walking speeds, we selected a lower (2–4 BL s^−1^) and a higher (8–10 BL s^−1^) speed range and transformed data from these speed ranges into the PC space we established for 5–7 BL s^−1^. We only allowed flies with 30 or more steps in a given speed range to be part of the analyses. Both additional datasets contained flies which had not been part of the dataset of 5–7 BL s^−1^ and vice versa. For all three datasets, we calculated the fraction of relative variability described by each PC. In addition, we compared how the walking behavior of all individual flies changed with walking speed in the dimensions of PCs 1–5 regarding their mean positions and standard deviations. A similar approach was taken with regard to the time a particular trial took place during an experiment (early versus late trials; for more detailed methods and results see Supplementary Materials and Methods and specifically, Fig. S6).

### Evaluation experiments

To test the suitability of the PCA-based approach for the description and analysis of idiosyncrasies in *Drosophila* walking behavior, we used an optogenetic approach. For this, the Gal4-UAS system was used to express GtACR1, an anion-selective channelrhodopsin, in a group of mechanosensory neurons in the legs. When activated optogenetically with green light, GtACR1 inhibits neurons expressing it (Govorunova et al., 2015; Mohammad et al., 2017). We used the transgenic *iav*-Gal4 line (O'Dell and Burnet, 1988) to target all chordotonal organs, including the femoral chordotonal organ (fCO), the largest sensory organ in the fly's legs. The resulting transgenic flies have previously been shown to exhibit a systematic and noticeable phenotype in walking behavior in an inhibition paradigm (Chockley et al., 2022). In contrast, wild-type control flies in that study (wild-type Berlin-K crossed to UAS-GtACR1) did not show a strong phenotype during phases of illumination with green light (see fig. S2 in Chockley et al., 2022). Replicating this previous study, flies walked spontaneously in the arena, initially in the dark (control condition, identical to the way in which the main dataset in the present study was collected). Once a fly had produced a valid trial, the green-light illumination was switched on (inhibition condition). After a valid trial was produced in the inhibition condition, the green-light illumination was switched off again; conditions were alternated in this way for the duration of the experiment.

We used these data to evaluate whether we could detect the known and strong fCO inhibition-specific effects in the PCA approach explored here and whether we could distinguish them reliably from putatively smaller stochastic effects based on general variability in walking behavior (these exist even between two randomly chosen samples from a larger dataset). For this, a minimum of 30 SCs each for dark (control) and light condition (inhibition) for individual flies was compared regarding the mean tarsal trajectories and the shift observed in the respective mean positions in PCs 2, 4 and 5. We tested the observed effect size for significance by comparing it with a bootstrap analysis performed on our original wild-type dataset that we established earlier for the speed range of 5–7 BL s^−1^: for 14 individual flies, we randomly selected two unique sets of 30 SCs each and compared these two sets with each other in the same way that we compared the two conditions (control and inhibition) for the transgenic flies.

### Symmetry axis in subspace of PCs 2, 4 and 5

The evidence that PCs 2, 4 and 5 describe interindividual differences in leg kinematics and posture (see Results) suggests that these PCs describe more general differences in the mean posture of individual flies. Among other aspects, this might refer to how sprawled the posture of an animal is or individual-specific distances between AEPs and PEPs, for instance. To give a comprehensible example, we systematically searched for an axis in the subspace of PCs 2, 4 and 5 along which the posture changes symmetrically with respect to the left and right body side. This axis was supposed to go through the center of the 3D subspace and respective postures were calculated from −5 to +5 times the standard deviation. Symmetry was measured by calculating the root mean square error (RMSE) between contralateral leg pairs after projecting the positions of one body side to the other. The axes with the highest symmetry scores and the respective postures are shown (see Results).

## RESULTS

We recorded 103 male flies of the wild-type strain Berlin-K with a total of 36,942 straight walking step cycles with a median of 242 step cycles per fly with 10th and 90th percentiles of 93.8 and 841 step cycles, respectively (minimum 28, maximum 1717). Eighty-eight flies yielded 30 or more step cycles within the speed range of 5 to 7 BL s^−1^ targeted in our analysis and thus were included in the PCA (for details, see Fig. S1). Restriction to this speed range was important, because many parameters of walking, such as duty cycle, stepping frequency, step amplitude or phase relationships, change systematically with walking speed. During evaluation experiments, we recorded 28 flies resulting from crosses between *iav*-Gal4 and UAS-GtACR1. They performed a total of 9694 step cycles in the control and inhibition condition with a median of 279.5 step cycles per fly with 10th and 90th percentiles of 108.9 and 726.6 step cycles, respectively (minimum 43, maximum 918). Fourteen of these flies produced 30 or more step cycles within the speed range of 5 to 7 BL s^−1^ for both conditions and were included in the analysis.

### Flies walk in an idiosyncratic manner

**Fig. 2.**
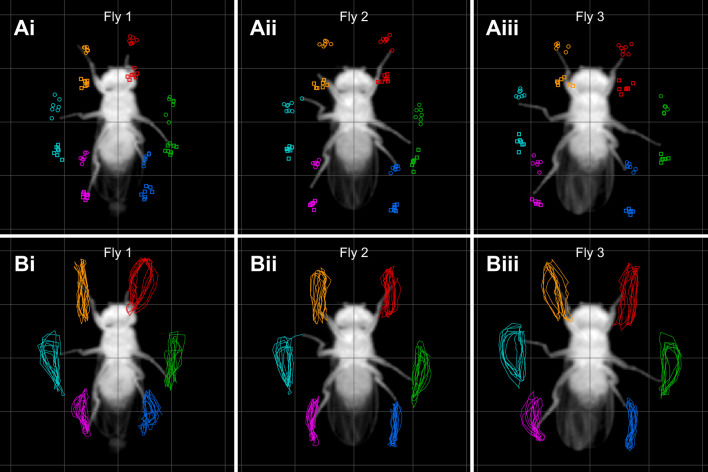
**Qualitative inspection shows flies walk in an idiosyncratic manner.** Here, interindividual variability of leg kinematics in three exemplary individuals is shown; i–iii refer to three different animals – each column is a different fly. (A) AEPs and PEPs of single walking bouts for three exemplary flies. Circles: AEPs; squares: PEPs. (B) Complete tarsal tip trajectories of the same walking bouts and flies as in A. Leg color coding: red, L1; green, L2; blue, L3; orange, R1; teal, R2; magenta, R3. These three flies are also referenced as exemplary flies in Figs 5 and 7.

Despite being all male, of identical age, from the same highly interbred wild-type strain, and tested under identical conditions, the flies analyzed here showed idiosyncrasies which were apparent in qualitative inspection. To illustrate these qualitative aspects and give a first exemplary impression of these idiosyncrasies that we would like to investigate further, Fig. 2 shows AEPs and PEPs (Fig. 2Ai–iii) as well as leg tip trajectories (Fig. 2Bi–iii) of three exemplary flies over the course of three individual walking bouts. Several distinguishing kinematic characteristics can be identified, such as (1) the overall width of the complete posture or leg pairs (compare Fig. 2Bi and Biii, for instance), (2) the straightness and shape of tarsal trajectories or (3) left–right symmetry. Qualitatively, it is also evident that intraindividual step-to-step (e.g. distance between AEPs and PEPs in one leg) variability is lower as compared with interindividual variability (i.e. difference in posture or asymmetries between Fig. 2Bi and Biii). These observations show that flies indeed walked idiosyncratically.

### PCA detects significant correlations of leg tip positions

**Fig. 3.**
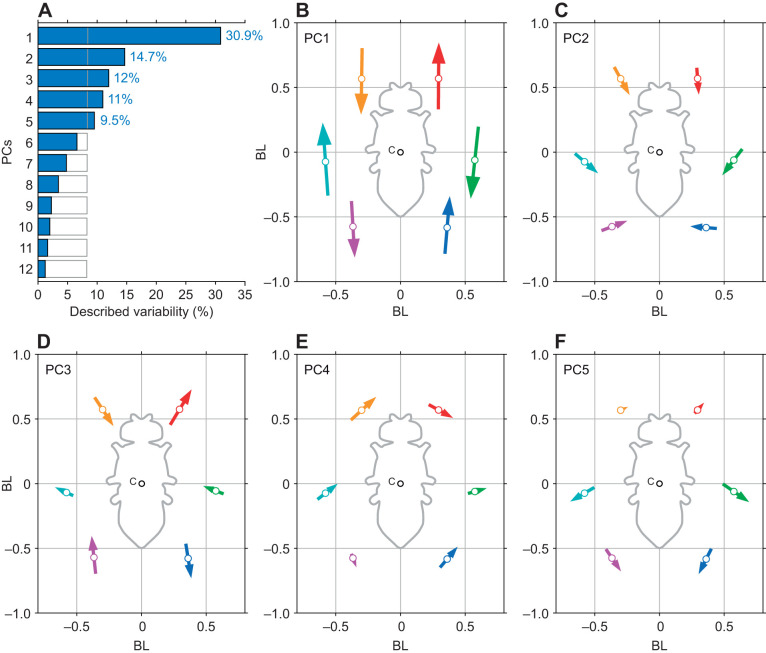
**Principal components analysis (PCA) finds significant correlations of leg tip positions.** (A) Fractions of variability in leg tip kinematics described by each principal component (PC). Filled blue bars: results based on PCA on the dataset acquired in this study (*n*=88 flies, *n*=30 steps per fly). Open bars: results for the same, but randomly permuted dataset (see Results). PCs 1–5 exceed the described variability expected based on the permuted dataset (respective percentages shown beside the bars). (B–F) Visualization of covariation of tarsal tip kinematics captured by PCs 1–5, respectively. White circles on the arrows depict the mean positions of all data points in the analysis. Arrow directions indicate the directions for a particular PC. Arrow lengths (magnitude) equal twice the standard deviation. As such, the contribution of each PC to the positioning of individual leg tips can be directly compared. The center of the body is indicated by the circle labeled C. Leg color coding: red, L1; green, L2; blue, L3; orange, R1; teal, R2; magenta, R3.

We used PCA to characterize linear covariations of leg tip movements. Such covariations either have a systematic biological basis or are random fluctuations. Here, the fraction of variability described by each PC for the randomized dataset approximated the inverse of the number of dimensions (∼8.33%). In contrast, fractions of variability described by PCs 1–5 for the original and unpermuted dataset exceeded this reference value, accounting for more variability than expected for randomized data (Fig. 3A), and described a cumulative ∼78% of the variability. For further analyses, we therefore focused on these first five PCs.

Here, each PC's loadings intuitively describe how 2D positions of the tarsal tips covary. This covariation can be a result of active leg movements during walking, i.e. protractions and retractions. Covariation in the two front legs, for instance, can be expected, because during anterograde movement of one front leg, the other one generally moves posterograde. Further covariation is based on general postural differences between individuals; how sprawled an individual's posture is, for instance, will affect its six legs fairly evenly and will be detected as positional covariation that describes the distance of the tarsi from the body. Individual PCs (i.e. their loadings) can be visualized as arrows to facilitate the comparison of the direction and magnitude (according to the amount of described variability) of covariations (Fig. 3B–F). PC 1 (30.9% described variability, Fig. 3B) captured the counter-directed anterograde and posterograde movements of neighboring legs, either ipsilaterally or contralaterally, and probably comprised the major component of forward locomotion. This PC suggestively groups the six legs into two groups (R1, R3 and L2; and L1, L3 and R2). The positions of legs in each of these two groups covary in the same direction and with the same magnitude, while the two groups have movement directions that are in exactly the opposite direction. We hypothesize that this covariation corresponds to an idealized tripod movement. However, the first PC described less than a third of the dynamics occurring in the leg tips. PCs 2 (14.7%) and 5 (9.5%) show shifts that are mirror symmetric along the longitudinal body axis for all three leg pairs. The directions of these covariations indicate that PCs 2 and 5 might describe interindividual differences in overall postural width (Fig. 2Ai and Aiii, for instance). PC 3 (12%) showed anterograde and posterograde directions of covariation for the front and hind legs (Fig. 3D). Here, however, the sign of covariation is negative on both body sides as compared with PC 1, and the middle legs do not display a comparable covariation in the direction of movement. Interestingly, in the context of inter-leg coordination, PC 3 would allow for a dissociation from the hypothesized strict tripod coordination as defined by PC 1 (but see next section for further elaboration). PC 4 (11%) only exhibits asymmetric covariations, mainly shifting the overall positions of the legs left and right; this is most pronounced in the front legs. While particularly this left and right covariation of the front legs in PC 4 (and to some extent of the other legs in PCs 2 and 5) intuitively gives the impression of curve walking, we found it to be an actual asymmetry between individuals which mainly resulted from differences in mean posture between flies and not from curve walking (compare front leg positions of fly 1 and fly 3 in Fig. 2Ai and Aiii). Fig. S2 shows quantitatively that flies that scored high or low on PCs 2, 4 or 5 did not produce reliable curve walking in either direction. Furthermore, we could not detect a strong relationship between the scores of PCs 2, 4 and 5 in a particular step cycle and the curvatures that were associated with that step cycle (Fig. S3A–C). The same holds for the relationship between curve walking and PCs 1 and 3 (Fig. S3D,E). Taken together, these interpretations of the loadings of PCs 1–5 tentatively suggest that these might describe aspects of inter-leg coordination (PCs 1 and 3) and posture (PCs 2, 4 and 5). In the following analyses, we present further evidence for this hypothesis.

### TCS reveals the relevance of PCs 1 and 3 for inter-leg coordination

**Fig. 4.**
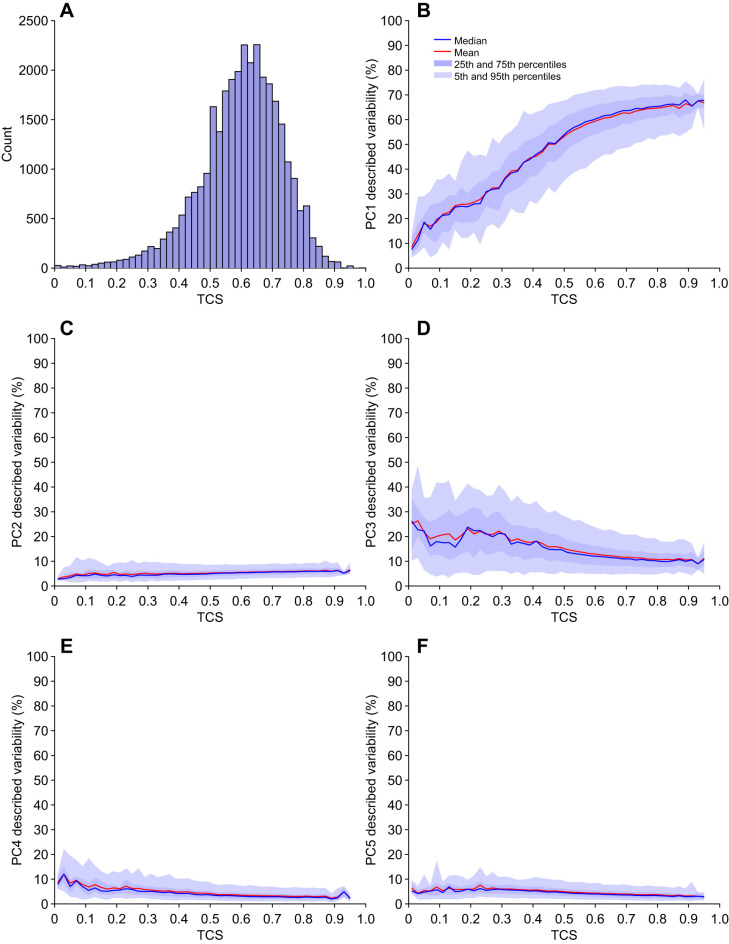
**Tripod coordination strength (TCS) reveals the relevance of PCs 1 and 3 for inter-leg coordination.** (A) Histogram of TCS values for all steps of all flies in the analysis pool (*n*=30,216). Note, that the majority of steps have TCS values in the range 0.5–0.75; this is largely because these TCS values are associated with walking speeds of around 6 BL s^−1^. The majority of flies tended to produce walking behavior at this speed (Fig. S1A,B). (B–F) TCS values plotted against described variability of PCs 1–5, respectively. TCS was calculated for consecutive cycles of both tripod groups. Scores for PCs 1–5 were determined by transferring the tarsal tip positions of respective steps into the PC space and calculating the relative described variability for each PC and each cycle. The higher the TCS, the more tripod-like a particular cycle is.

Using TCS as a measure for how tripod-like a stepping sequence is, we tested for correlations between inter-leg coordination patterns and scores of individual PCs. Insects use a speed-dependent continuum of inter-leg coordination patterns (Szczecinski et al., 2018; Wosnitza et al., 2012). High walking speeds are associated with the so-called tripod coordination, slower walking speeds effect coordination that deviates from tripod coordination (Szczecinski et al., 2018). Canonical tripod coordination (rarely observed in its ideal form) thereby corresponds to anti-phasic activity of two tripod leg groups (sets of ipsilateral front and hind legs and the contralateral middle leg). In this context, TCS is a single value that describes the similarity between a particular coordination pattern and ideal tripod coordination (see Wahl et al., 2015; Wosnitza et al., 2012). We used TCS to test whether covariations described by PCs were indeed correlated with certain coordination patterns. We first calculated TCS for all instances in which complete leg cycles of a particular tripod group were available. Then, where possible, we averaged the TCS values of two subsequent tripod cycles, effectively extending the TCS definition from single tripod groups to all six legs. The values calculated this way contained steps from the complete dataset of 103 flies (all walking speeds) and yielded 30,216 instances of two complete and subsequent tripod cycles (Fig. 4A). The distribution of TCS values generally reflected the distribution of walking speeds; TCS values were predominantly found in the range between 0.5 and 0.75 and were associated with walking speeds around 6 BL s^−1^ (see Fig. S1A,B).

Leg tip positions within the respective interval were mapped into PC space and relative fractions of variability described by each PC for this subset of data were calculated. The fraction of variability described by PC 1 was found to be strongly positively correlated with TCS (Fig. 4B). Conversely, the described variance of PCs 2, 4 and 5 did not show a clear correlation with the respective TCS values (Fig. 4C,E,F). PC 3 was negatively correlated with the TCS values, but this was less pronounced than for PC 1 (Fig. 4D). Taken together, these results suggest that PCs 1 and 3 describe a substantial part of the dynamic and coordination-related aspects of walking behavior. In contrast, PCs 2, 4 and 5 seem to describe features that are independent of these dynamic aspects; we explore their relevance in greater detail in the following sections.

### PCs 2, 4 and 5 describe interindividual kinematic differences

**Fig. 5.**
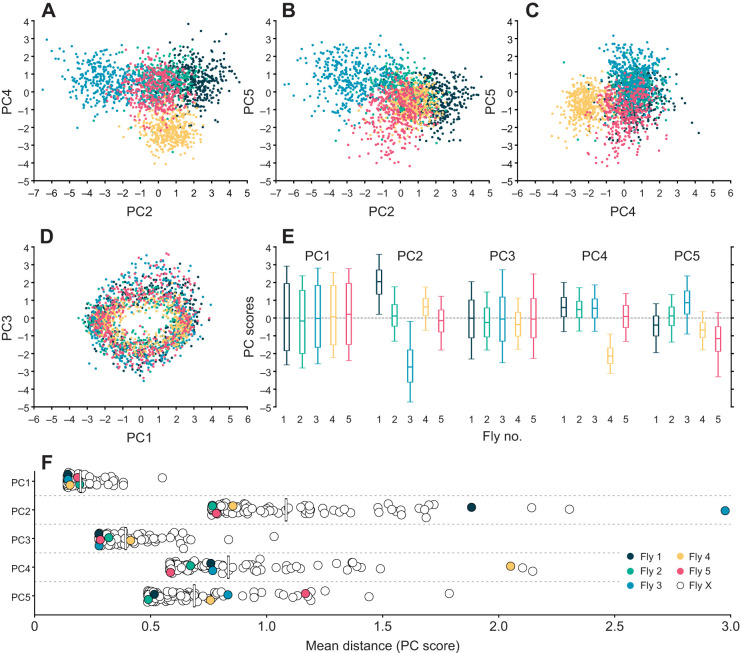
**PCs 2, 4 and 5 describe interindividual differences of leg kinematics.** Subspaces spanned by (A) PCs 2 and 4, (B) PCs 2 and 5, (C) PCs 4 and 5, and (D) PCs 1 and 3. Different colors correspond to individual flies. Each dot represents a fly's posture at a given time, selected randomly from the data that contributed to the analysis (*n*=400 per fly). Note the difference between the distributions in A–C and that in D. Also note the systematic elliptical shape of the distribution in D (see Movies 2 and 3). (E) Boxplots (median, upper and lower quartiles and 5th and 95th percentile) indicating the distribution of scores of the five flies in A–D (same color code) for the first five PCs. Flies 1, 2 and 3 refer to the data of the exemplary flies presented in Fig. 2. The data for flies 4 and 5 have been added to further populate and exemplify the subspaces in A–D. See Fig. 6Aii–Dii for summarized data of all flies (*n*=88) and Movie 1 for an animation of a plot of all data, analogous to A–D in this figure. (F) Mean distance of each fly to all other flies along the dimensions of the first five PCs. Flies 1–5 are color coded in the same way as for A–E. Fly X refers to arbitrary fly other than flies 1 to 5. Mean values for each PC are depicted as vertical lines. Note that flies cluster more in PCs 1 and 3, indicating higher similarity in these PCs (see also Fig. 6Dii).

Individual flies can be compared in subspaces spanned by the first five PCs to check whether interindividual differences of fly walking behavior are described by these PCs. To do this, and as a first step, we plotted data from five exemplary flies in 2D PC subspaces (Fig. 5A–D), spanned either by pairs of PCs 2, 4 and 5 (Fig. 5A–C) or by PCs 1 and 3 (Fig. 5D). The subspaces spanned by PCs 2, 4 and 5 show relatively clear separation between flies (Fig. 5A–C), while in PCs 1 and 3 (Fig. 5D) the data largely overlap (for the complete dataset of 88 flies, see Movie 1). Interestingly, data plotted in Fig. 5D form an elliptic ring around the origin, indicating that they describe fundamentally different aspects of walking. We found that over the course of a step cycle, the postural representation in this 2D subspace spanned by PCs 1 and 3 cycles the origin once (Movies 2 and 3). The dimensions of this ring were largely invariant for individuals, but depended on inter-leg coordination. Fig. 5E confirms that all five flies had individual signatures regarding their scores for PCs 2, 4 and 5, while scores for PC 1 and 3 were comparatively similar (median values for PC 1 and 3 were approximately zero for these flies). Extending this analysis to the complete dataset (Movie 1), we found that the average distances between flies in the dimensions defined by the five PCs was larger in PCs 2, 4 and 5 than in PCs 1 and 3 (Fig. 5F). These results demonstrate that PCs 2, 4 and 5 describe some of the interindividual differences, while the absence of separation in the subspace spanned by PCs 1 and 3 shows that these describe features that are largely invariant between individuals (such as inter-leg coordination or the basic movement of legs).

### Influence of walking speed and recording time

**Fig. 6.**
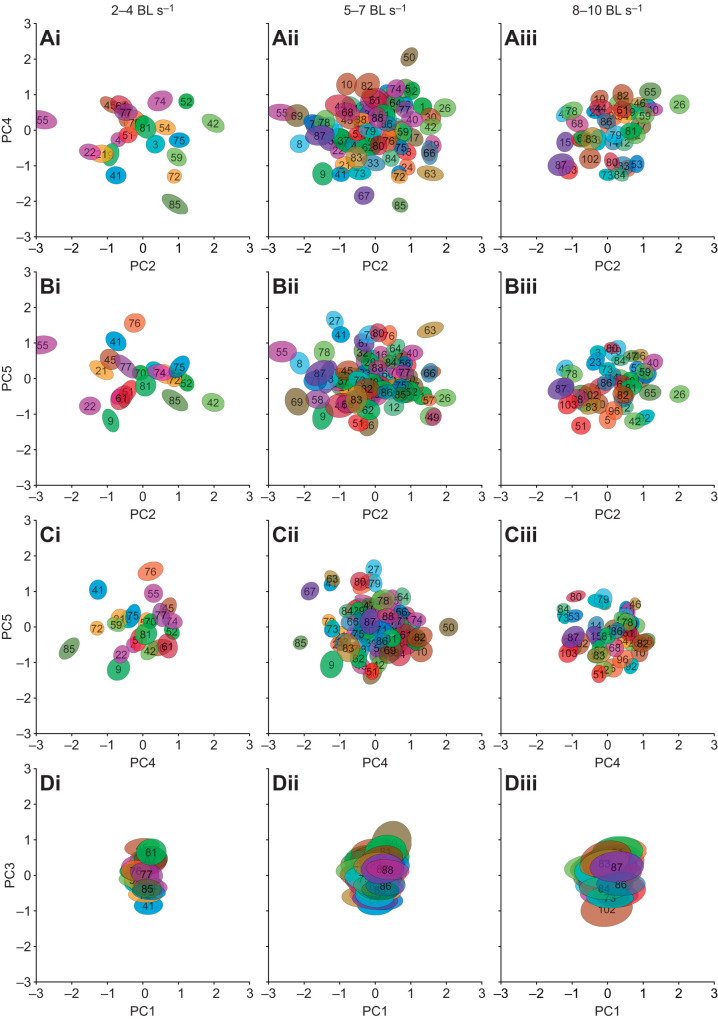
**Data distribution of flies for three different speed ranges in the subspaces defined by PCs 1–5.** Instead of individual data points, as in Fig. 5A–D, all data points are depicted here in the form of ellipses that describe the bivariate distribution of the underlying dataset for each fly (0.3 times the standard deviation). Left column (i): 2–4 BL s^−1^, *n*=23 flies. Middle column (ii): 5–7 BL s^−1^, *n*=88 flies. Right column (iii): 8–10 BL s^−1^, *n*=43 flies. (A) Subspace of PCs 2 and 4, corresponding to Fig. 5A. (B) Subspace of PCs 2 and 5, corresponding Fig. 5B. (C) Subspace of PCs 4 and 5, corresponding to Fig. 5C. (D) Subspace of PCs 1 and 3, corresponding Fig. 5D. Note, that the spread of the data in D (PCs 1 and 3) is much smaller than that in A–C and that most flies occupy the same position in this subspace (also compare Fig. 5D and Fig. S5). The changes in mean position of flies for different walking speeds are depicted in Fig. S5.

To investigate how walking speed affects aspects of inter-leg coordination and idiosyncrasies of walking, we analyzed additional subsets of data from different speed ranges in the original PC space. We selected the range between 2 and 4 BL s^−1^ and 8 and 10 BL s^−1^, to extend the analysis to slower and faster walking speeds, respectively. Because fewer steps were recorded for these speeds (see also Fig. S1A,B), fewer flies were included in these analyses; several of these flies had not been part of the original PCA. Nevertheless, for the lower speed range we found 23 flies and for higher range we found 43 flies with 30 or more steps. Fig. S4 shows how described variability changed for the lower (Fig. 6Ai–Di) and higher (Fig. 6Aiii–Diii) ranges of walking speeds compared with the original speed range (Fig. 6Aii–Dii, also compare Fig. 3A). The fraction of described variability of PC 1 strongly increases by approximately 21% from the low to the high speed range, while PC 3 increases by approximately 3.4%. In contrast, fractions of described variability for all other PCs decreased, resulting in the accumulation of described variability in PC 1 and PC 3. This accumulation of PCs 1 and 3 and the reduction in PCs 2, 4 and 5 suggests that the overall variability of the flies' walking behavior decreased with increasing walking speeds.

If idiosyncrasies are constant and can be described by PCs 2, 4 and 5, as we hypothesize here, flies should vary strongly with respect to each other in this space but should keep their individual positions when walking speed changes. To investigate this, we characterized the average positions and distributions of all individual flies in PC space separately for all three speed ranges and how their positions changed between speeds (Fig. 6). Analogously to the exemplary flies in Fig. 5, the complete population of flies varies strongly in the space spanned by PCs 2, 4 and 5; this is true for the original speed range (Fig. 6Aii–Cii), as well as for the lower (Fig. 6Ai–Ci) and higher range (Fig. 6Aiii–Ciii), supporting the notion that idiosyncratic aspects of walking are mainly described by these PCs, even when considering different speeds. In combination, Fig. 6A–C shows that most flies can be distinguished from others in their position with regard to at least one of these three PCs. In contrast, the data for individual flies overlap strongly in the space spanned by PCs 1 and 3 (Fig. 6D); at the same time, each individual fly's data distribution (ellipses in Fig. 6) increases in the direction of PC 1 with walking speed, suggesting an increase in the importance of this PC, hypothesized to be reflective of tripod coordination. With increasing walking speed, the average distance from any fly to all others in the subspace of PCs 2, 4 and 5 decreases from 1.87 to 1.75 to 1.54 (also compare Fig. 5F). In the subspace of PCs 1 and 3, we measured average distances of 0.51, 0.47 and 0.49, respectively. Not only are the average distances between flies generally much larger for PCs 2, 4 and 5 than for PCs 1 and 3 but also they are more strongly affected by increasing walking speed. Most importantly here, however, is the relative constancy of positions in the space of PCs 2, 4 and 5 (see Fig. 6 for absolute positions and Fig. S5 for shifts). This indicates that the kinematics described by these PCs largely remain constant for an individual, even when that individual changes its walking speed. Together with the clear identifiability of individuals in this space, this suggests that these PCs indeed describe individual aspects of walking that are independent of walking speed.

### Optogenetic inhibition demonstrates the descriptive potential of PCs

An optogenetic inhibition experiment was performed to evaluate the potential of PCs 2, 4 and 5 to describe systematic differences in posture and leg positioning. We crossed the *iav*-Gal4 driver line with UAS-GtACR1. The resulting F1 generation expressed the inhibitory channelrhodopsin GtACR1 in the neurons of chordotonal organs, including the fCO. Previous experiments have shown that inhibiting these sensory structures causes systematic changes in walking kinematics (Chockley et al., 2022). Here, we used these expected changes to test whether they can be detected within the subspace spanned by PCs 2, 4 and 5. We focused on this particular PC subspace because the changes due to inhibition of the fCOs during walking were found to be largely postural (Chockley et al., 2022) and, as we have shown in the preceding analyses (Figs 4, 5 and 6), this subspace seems to capture postural changes well.

**Fig. 7.**
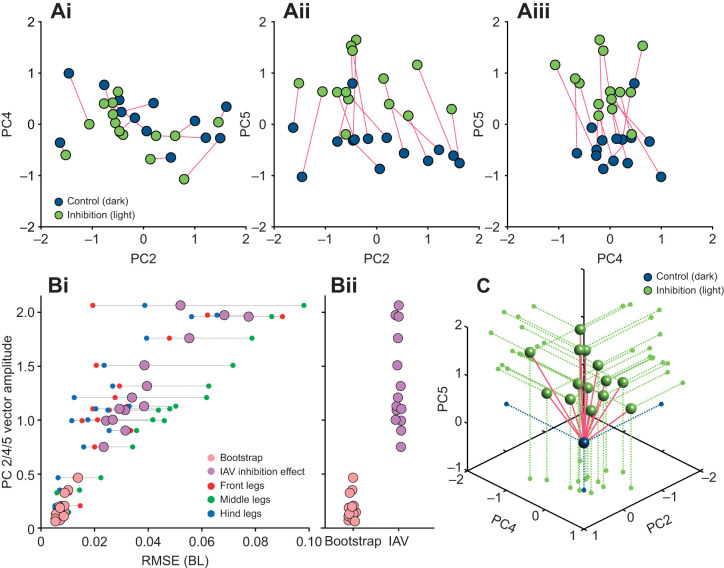
**An optogenetic inhibition experiment demonstrates the descriptive potential of PCs.** (Ai–iii) 2D representations of the inhibition effect in subspaces spanned by combinations of PCs 2, 4 and 5 [blue, steps in the dark; green, steps in green light (femoral chordotonal organ, fCO, inhibited)]. Red lines connect mean positions of individual flies in the two conditions. (Bi,ii) Vector amplitudes (i.e. effect size) of the inhibition experiments and the bootstrap analysis plotted against the root mean square error (RMSE) of the leg tip trajectories. Pink circles depict results for the bootstrap analysis, purple dots are those for inhibition experiments (IAV, inactive). Red, green and blue circles indicate the RMSE for front, middle and hind legs. (C) 3D representation of the inhibition effect (green) relative to the control condition (blue). For clarity, control conditions were set to the origin.

Because of the alternating darkness/light paradigm (see Materials and Methods), each individual fly produced trials in the dark (corresponding to wild-type condition) as well as during green-light illumination (corresponding to inhibition); each fly therefore served as its own control. For each fly with more than 30 steps in the control (dark) and the inhibition condition (light), the mean positions for both conditions were analyzed in the subspace spanned by PCs 2, 4 and 5 (Fig. 7Ai–Aiii). The length of the vector describing the difference between the dark and light condition was compared with a bootstrap analysis. For this, two sets of 30 steps each were randomly drawn from single flies of the original dataset and plotted in the same way as the data from the inhibition experiments (Fig. 7B). The results for the inhibition experiments showed no strong preference for shift directions for PCs 2 and 4, but a clear and consistent shift towards more positive values for PC 5 (Fig. 7Ai–iii). In contrast, the bootstrap analysis expectedly resulted in more random shift directions whose magnitude was also smaller. The mean effect sizes measured for PCs 2, 4 and 5 (i.e. vector lengths) were plotted against the observed differences in the mean leg tip trajectories between control and inhibition, expressed as RMSE, showing a positive correlation (Fig. 7Bi); the stronger the difference between control and inhibition on the level of leg tip kinematics, the larger the shift in PC space. The difference in RMSE was mainly driven by the middle legs (green circles), matching the effect described by Chockley et al. (2022). Fig. 7Bii depicts the vector lengths for the inhibition experiments and the bootstrap analysis, showing that effect size was much larger on average for the inhibition experiments. PC 2 shows shifts to more negative values for 10 individuals, but also four in the opposite direction, although with much smaller amplitudes (Fig. 7Ai–Aii). PC 4 shows even smaller and less consistent effects, probably because the inhibition of fCOs affected the two body sides equally, while PC 4 describes only asymmetric covariations (Fig. 3E). However, the clear and consistent shifts from the dark to light condition in PC 5 demonstrate that PCs in general can be used to compare and quantify effects and their magnitude in a reduced number of dimensions.

### A symmetry axis demonstrates how individual postures are encoded in PCs 2, 4 and 5

**Fig. 8.**
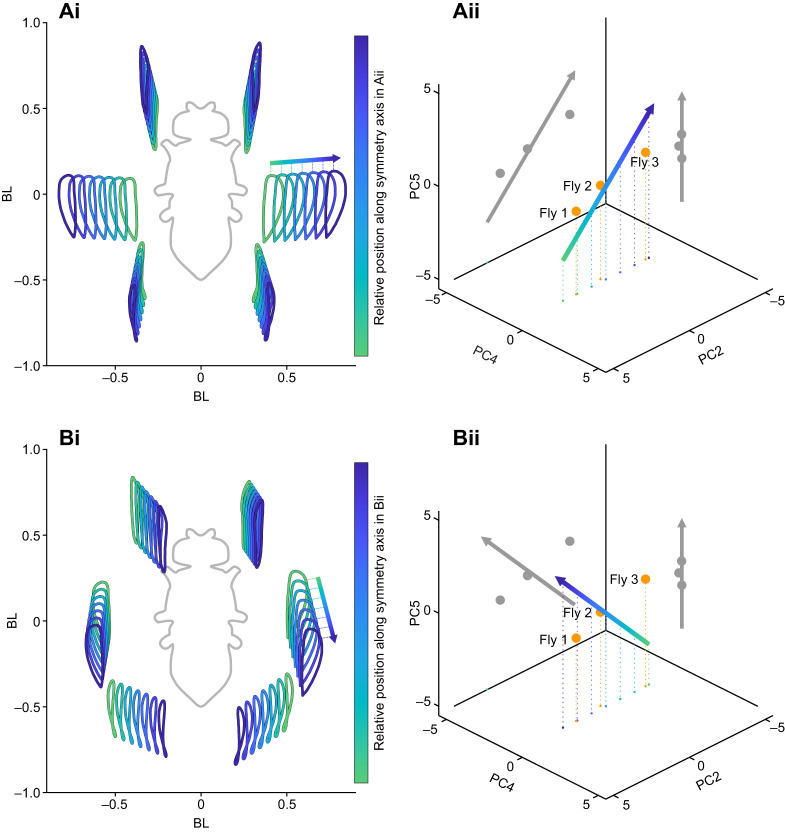
**A symmetry axis demonstrates how individual postures are encoded in PCs 2, 4 and 5.** Leg tip trajectories for different points on the symmetry axis (Aii,Bii) in the subspace of PCs 2, 4 and 5 describing systematic differences in postural width (Ai,Bi). Color code in Ai and Bi corresponds to color shading along the axis in Aii and Bii, respectively, and is additionally shown in the form of a gradient bar on the right of Ai and Bi. Orange circles depict the mean positions of flies 1, 2 and 3 introduced in Fig. 2 and also referenced in Fig. 5. Gray circles and arrows are projections of the 3D sequence of explored combinations into 2D subspaces spanned by PCs 2 and 5 as well as PCs 4 and 5, respectively.

To further explore which interindividual differences might be encoded by PCs 2, 4 and 5, we systematically searched for an axis in this PC subspace that reflected symmetric postural changes with regard to the longitudinal axis of the body. We found an axis which described the mean distance of the leg tips to the fly body (Fig. 8Aii). The contribution of PC 4 was very weak, which is not surprising as we searched for highest symmetry and PC 4 describes asymmetric covariations. Interestingly, flies 1–3 (referred to in Figs 2 and 5) are close to the symmetry axis and the qualitative differences in their postures, which mainly involved how sprawled these flies walked, are represented well in the artificial postures shown in Fig. 8Ai. PC 4, in contrast, seems to describe less symmetric differences between individuals as presented for fly 4 in Fig. 5. To additionally demonstrate how PCs 2, 4 and 5 might describe differences in posture, we constructed an axis that was perpendicular to that shown in Fig. 8Aii. The resulting axis was still mainly co-planar with the subspace of PC 2 and 5 (Fig. 8Bii). The respective changes of fly posture displayed in Fig. 8Bi are almost perpendicular to those in Fig. 8Ai, mainly shifting the average leg positions anterograde and posterograde. Together, these two PCs compactly describe a large range of different, but symmetrically arranged fly postures. Interestingly, PC 4 did not show up in our unbiased approach to find these symmetry axes. This is unsurprising, when we consider that this PC largely describes left and right shifts in leg position (Fig. 3E). Together with PCs 2 and 5, it is then possible to more precisely describe the left–right asymmetry and, because of the linear nature of PCA, PC 4 would simply shift the posture accordingly.

## DISCUSSION

Here, we used a dataset of a large number of flies and many instances of walking sequences to investigate the naturally occurring intraindividual and interindividual variability of walking on the level of leg tip kinematics. Initial qualitative observations readily replicated anecdotal impressions from previous studies that flies walk in an idiosyncratic manner (Fig. 2). A quantitative analysis using PCA revealed that much of the observed kinematic variability can be captured by a lower-dimensional representation of five PCs (Fig. 3A). Two principal components (PCs 1 and 3) captured general variability aspects across individuals related to invariant movement patterns, such as the general positional changes during a step cycle and inter-leg coordination (Figs 3B,D and 4B,D); at the same time, three other PCs (2, 4 and 5) described individual-specific aspects of walking (Figs 3C,E,F, 5 and 8). PCA was able to separate these two different sources of variability into sub-domains (Figs 5 and 6Aii–Dii) and the contributions of the individual-specific PCs can be regarded as a fingerprint of a fly's idiosyncrasies during walking (Figs 5E, 6Aii–Cii) which are distinct from more general features shared by all flies. Investigation of different ranges of walking speeds, as well as the comparison of trials recorded at different times during experiments, showed that idiosyncratic aspects of walking are largely constant for an individual, while general invariant dynamics of walking, as captured by PCs 1 and 3, increased in significance with an increase in walking speed (Fig. 6 and Figs S4 and S5). Exploration of the individual-specific PCs 2, 4 and 5 revealed that general high-level features, such as kinematic changes after experimental interventions (Fig. 7) or overall posture of tarsal tips (Fig. 8), can be compactly detected, described and quantified.

Postural variability in the present data occurs on the intra-step level, the step-to-step level and the interindividual level. The intra-step level thereby refers to positional variation of the tarsal tips over time, essentially the periodic back and forth movement of legs during protraction and retraction. The specifics of these movements are mainly captured by PCs 1 and 3, with PC 1 reflecting the canonical tripod coordination (Fig. 3B) and PC 3 reflecting deviations from this tripod coordination. Because this dynamical component of variability is large as compared with more subtle and static postural aspects, it is not surprising that PC 1 is the most prominent PC and generally captures a large part of the variability across individuals. Together with PC 3, it therefore is suited to compactly represent the overall coordination an animal uses. Previously, there have been only a few attempts to compactly characterize coordination of the six legs of walking insects; TCS is one measure (Wahl et al., 2015; Wosnitza et al., 2012), but it has limitations, especially if the walking pattern deviates strongly from tripod coordination. A characterization based on these two PCs, whose contributions also seem to be negatively correlated with each other (see Fig. 4B,D), could serve as an alternative approach to efficiently describe coordination in future studies. An aspect that was not analyzed in detail here is the temporal variation of the contribution of these two PCs, their score time courses. As PC 1 and 3 captured the actual movement of the legs, the scores of these two PCs will generally be modulated periodically over the time course of the stepping movements of individual legs (Movies 2 and 3). Further examination in this regard could reveal more subtle relationships between how PC 1 and 3 are modulated in the context of inter-leg coordination and how individual flies combine these two patterns to establish tripod coordination (PC 1) and deviations from it (PC 3).

In contrast, PCs 2, 4 and 5 capture more static and interindividual postural differences (Figs 4, 5, 6 and 8). The postures of individual flies occupy different parts of this PC subspace. Consequently, this subspace is helpful to describe these individual differences in the first place, but this can be readily expanded. We explored several exemplary expansions here (see discussion for walking speed-related results below). The first was an experimental intervention that introduced known changes in leg kinematics which, in turn, were picked up clearly in the space spanned by PCs 2, 4 and 5. The second expansion was a top-down search in this space that describes overall changes in postural width. This approach can be useful in other novel interventions, in which putative effects are to be detected but for which a more unbiased analysis is desirable or in which several effects combine more subtly. Previous studies focused on kinematic parameters such as stance amplitude, durations of swing or stance phases, or AEPs and PEPs (Mendes et al., 2013; Wosnitza et al., 2012; Strauß and Heisenberg, 1990; Ramdya et al., 2017). While these singular measures are informative, a more unbiased way of looking at putative effects of interventions might reveal other effects that are less intuitive, more complicated or interdependent.

PCA has the general limitation of only capturing linear correlations between analyzed variables. It is conceivable that more complicated approaches for dimensionality reduction will yield more compact or clearer description and separation of the different levels of behavioral variability. A previous study, for instance, used Uniform Manifold Approximation and Projection (UMAP) to explore high-dimensional kinematics of walking flies and found similar reductions of these data (DeAngelis et al., 2019). However, the analysis we use here captures many interesting and helpful aspects of variability; the strength of PCA in the present context is its intuitive interpretability with regard to what individual PCs mean for kinematics.

Considering the putative roles of the five PCs, with 1 and 3 responsible for aspects related to inter-leg coordination and 2, 4 and 5 responsible for postural aspects, we can speculate that a particular combination of all of these in an individual has implications for how walking is controlled, especially with regard to static stability (Szczecinski et al., 2018). Stability is dependent on the duration of ground contact of the legs as well as their positioning in relation to the center of mass. This might directly relate back to the individual PCs. Based on the findings presented here, we assume that the ratio of PCs 1 and 3 mainly determines inter-leg coordination (Movies 2 and 3) and that the remaining PCs determine overall posture (including AEPs and PEPs). Thus, from a motor control perspective, we speculate that PCs 1 and 3 might be dynamically mixed together, based on the intended inter-leg coordination and walking speed, but also on aspects of posture that have become fixed in ontogeny, such as its symmetry. High walking speeds require exact alternation of the swing and stance phases of neighboring legs, in the sense of stability. More asymmetric overall postures (e.g. in flies that score high on PC 4, for instance) might therefore necessitate an earlier switch to more tripod-like and therefore more stable coordination patterns (as reflected in strong contribution of PC 1) to compensate for deviations from perfect symmetry.

Importantly, the first analysis in the present study focused on straight walking in a narrow speed range and on male flies of the same age, reared in identical conditions, and from the same highly interbred wild-type strain. These restrictions were intentional, as we wanted to first establish the general approach and its usefulness for a more controlled subset of all possible behavioral data. Therefore, we wanted to exclude additional sources of potential variability, based on parameters such as walking speed, age or sex. Even in this relatively controlled dataset, we nevertheless found a diversity of idiosyncratic ways of walking. But it is true that this first analysis necessarily only reflected kinematic details which were contained in the selected data and the conclusions, particularly with respect to the constancy of idiosyncrasies, could be limited.

We therefore extended the analysis to different walking speeds. Walking speed has strong effects on many kinematic parameters and inter-leg coordination in walking insects (DeAngelis et al., 2019; Strauß and Heisenberg, 1990; Szczecinski et al., 2018; Wosnitza et al., 2012). This analysis showed that idiosyncrasies remain largely constant when individuals change walking speed, but that aspects of inter-leg coordination change to some extent. In particular, PC 1 becomes more important, a finding that is in line with previous established results that coordination becomes more tripod-like the faster a fly walks (Szczecinski et al., 2018; Wosnitza et al., 2012). Another interesting observation (until now anecdotal) relates to how variability of walking changes as a function of walking speed. These observations suggest that AEP and PEP positioning or the phase relationships between legs, for instance, become less variable at higher walking speeds. Our results now support this observation in a quantitative manner: flies tend to become relatively more similar to each other with increasing walking speed, i.e. the described variability of PCs 2, 4 and 5 (interindividual variability) decreases and PCs 1 and 3 (aspects of inter-leg coordination) increase in importance. While the exact reason for this is unknown at this point, we hypothesize that it could be related to the stability requirements of fast walking; a previous study suggests that the region in phase space associated with stable walking at high speeds is smaller than that for low speeds (Szczecinski et al., 2018) and that fast walking necessitates tripod coordination. Flies (1) should therefore walk in a less variable way at these speeds and (2) should also become more similar to each other in walking. Our results support this notion and future studies will investigate this aspect more closely.

Other natural extensions of this analysis might focus on the influence of curve walking or age, among others. Expanding the number of these factors a controlled way should successively elucidate the full spectrum of natural variability in fly walking behavior, thereby enabling more precise investigation of the fundamental principles underlying the motor control of insect walking. Even stronger changes in the specific makeup of PCs and their contributions are expected when not only straight but also curve walking enters the dataset. Curve walking entails pronounced changes in the kinematics of all legs as indicated by previous studies, mainly on stick insects (Dürr and Ebeling, 2005; Gruhn et al., 2008). Legs contribute in a specific kinematic manner to curve walking. These changes will be reflected in the detected PCs, adding another layer of variability that reflects invariant aspects of curve walking, on the one hand, and potential interindividual differences in the way single flies implement this change, on the other.

Here, we provide the first extensive study of variability on the kinematic level in *Drosophila* walking behavior in genetically very similar and highly interbred individuals. The combination of dimensionality reduction using PCA, the inter-leg coordination measure TCS and inhibition experiments sheds some light on the natural variability present in walking behavior and suggests an enhanced way of characterizing data of freely walking flies. We thereby hope to build the foundations for further systematic investigation of the principles underlying the natural variability of walking.

## Supplementary Material

10.1242/jexbio.247878_sup1 Supplementary information
