# Supplementary material for: Natural variability and individuality of walking behavior in Drosophila
Source: J Exp Biol. 2024 Nov 21;227(22):jeb247878. doi: 10.1242/jeb.247878 (PMC11607691; doi:10.1242/jeb.247878)
Supplement: Supplementary information [file jexbio-227-247878-s1.pdf]

# Supplementary Materials and Methods

## Speed distribution across all recorded flies

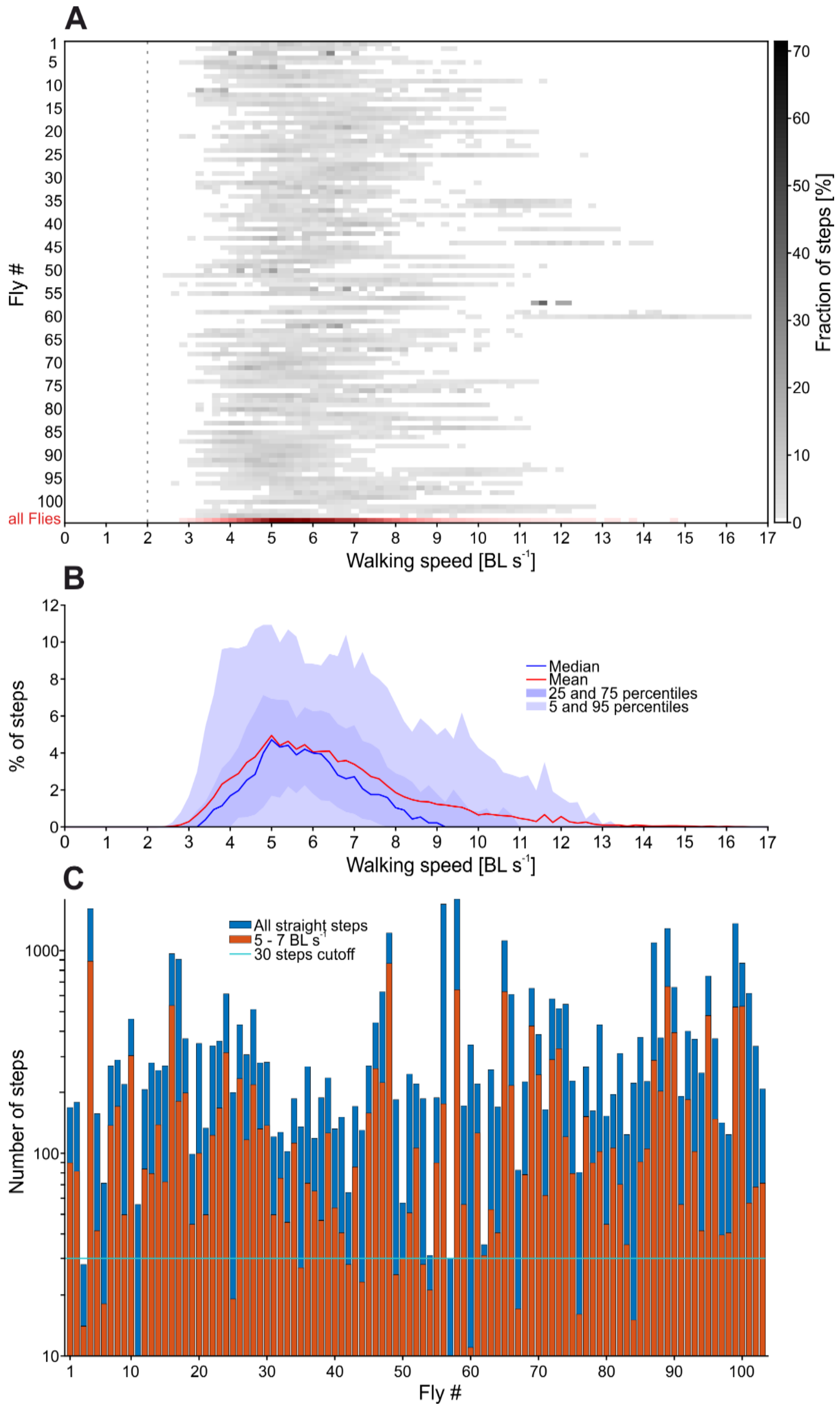


Journal of Experimental Biology • Supplementary information

**Fig. S1.** Distribution of walking speeds across all recorded flies and step cycles. (A) Each row shows one fly. Bin size for walking speed was set to 0.2 BL $s^{-1}$. Walking bouts below 2 BL $s^{-1}$ were not recorded. Last row (red) shows the total walking speed distribution for all flies and 36,942 recorded step cycles. (B) Distribution of walking speeds for all flies, same data as in A (last row). (C) Logarithmic histogram of the number of step cycles for all recorded flies. Blue = total number of straight step cycles; orange = number of step cycles between 5 and 7 BL $s^{-1}$; teal = cutoff line for flies which were taken into the analysis pool at 30 step cycles.

Figure S1 displays the distribution of walking speeds for all flies and all step cycles pooled together (see line labeled “all flies”). Some flies tend to walk faster than others; most of them, however, preferred walking speeds between 5 and 7 BL $s^{-1}$, which is why this speed range was selected for the study. Line total indicates that walking speed across steps tends to be log normal distributed. No steps associated with walking speeds close to the cutoff point at 2 BL/s were recorded. Of the 103 recorded flies, 88 produced 30 or more steps cycles in the target speed range of 5 to 7 BL $s^{-1}$.

## Automated detection and annotation of flies

While detection and annotation of the flies was fully automated, each video was manually checked for annotation errors and corrected if necessary. The detection and annotation process was structured as follows. For each image frame in a video, we created a binary image with a simple threshold operation, i.e. bright pixels above the threshold became white, dark pixels below the threshold became black. To remove small regions of white pixels we used a morphological opening operation (equivalent to an erosion operation, followed by a dilation, MATLAB function *imopen.m*). After this operation, only the region of white pixels corresponding to the fly was left. The position of the fly was then detected as the mean position of this remaining blob. This position was used to crop a square region containing the fly from the image. The size of this cropped region was adjusted to the fly’s length. Using DLC, we then detected the neck and abdomen in this cropped subregion and used this information to rotate the subregion such that the fly’s longitudinal axis was aligned in parallel to the y-axis of the image. In two subsequent runs of DLC we then detected the leg tips on the right body side, as well as those on the left side, respectively (see also Fig. 1B).

## Discrimination of swing and stance phases

Transition points between swing and stance phases, i.e. lift-off and touchdown, were automatically determined by analyzing the movement of the markers (i.e. their absolute speed) in the arena-centric coordinate system of the original video. For every marker and every frame, the distance between the current marker position to the position in the subsequent frame was calculated. Due to noise the exact location of a marker slightly varies from frame to frame independently of actual leg movement. Therefore, a threshold had to be defined to discriminate frames with legs moving relatively to the ground from frames with legs having ground contact. An empirically determined threshold of 1.5 pixels per frame produced precise and reliable results and was used for the complete analysis in this study. Additional conditions were defined to further increase the robustness of the algorithm. For example, a swing phase had to last at least 3 frames (equal to 15 ms), otherwise it was not detected as such. Additionally, the correct automatic determination of lift-offs and touch-downs was reviewed manually during the process of screening for errors in the DLC annotations.

## Training of DeepLabCut

Automated annotation of all six leg tips, the neck and the tip of the abdomen was carried out with DeepLabCut (DLC), a deep neural network approach (Mathis et al., 2018). DLC was trained on exemplary frames of freely walking fruit flies along with manually generated information about the locations of the eight body parts of interest (neck and abdominal tip, as well as the tips of all six legs). Respective training data was produced by manually annotating video frames in a custom-written MATLAB program.

Coordinates of the body parts of interest were stored together with the cropped video in a single MAT-file. For the initial training data set, 500 frames were collected from ten flies in various positions. Training data sets were digitally augmented by randomly applying rotations, translocations, and resizing. This prevented adaptation of the network to a certain orientation, size, or position in the video frame. Rotations were not applied for the training of the networks which were used for leg tip detection, since the fly body axis was aligned vertically after the

Journal of Experimental Biology • Supplementary information

detection of neck and abdomen. Here, complexity of possible inputs was reduced to potentially increase accuracy and reliability of the automated annotation.

Even though the arena surface was cleaned regularly, small pieces of dust or debris accumulated on it to some extent. These pieces typically can have sizes and shapes that make them similar in appearance to the tips of the fly's legs and were sometimes detected as such. To make the trained networks more robust to these pieces, randomly generated, virtual specks of dust and debris were added to each training frame. Modifications of training frames were carried out in a custom-written MATLAB program. The same program was used to create training directories, which contained all files necessary for training of DLC. Training was run for 400 thousand to 1 million iterations, taking 8 to 20 hours.

**Selection of straight segments**

The recording software triggered data acquisition dependent on the length of the trajectory rather than its shape. Hence, the acquired video data needed to be scanned for segments of straight walking. For thousands of videos it is fairly labor-intensive to search for such segments manually, isolate them from the remaining video, and store them in another directory. Moreover, to what extent a trajectory is considered to be straight is subjective and the criteria may change unconsciously over time. Therefore, an algorithm judging each trajectory by objective criteria was considered to be the best option. Since all further analyzed trajectories should be of certain straightness while losing as few frames as possible, two approaches for detecting straight segments were combined to compensate for their individual disadvantages. For both approaches, the original trajectory was first divided into equidistant pieces of 0.5 mm length. One approach extracted the angles between neighboring pieces as an estimator for straightness, in a way that two or more pieces of trajectory with an angle of 5° or less to each other were considered to be straight. The other approach used the quintic spline function in MATLAB to fit a spline to equidistant points. Each point with its two direct neighbors can be used to define a circle with a radius correlating to the curvature at this point of the trajectory. An infinite radius would mean that all three points are on a perfect straight line. The smaller

Journal of Experimental Biology • Supplementary information

the radius becomes, the higher the curvature is. Constellations points with an inverse radius of of 0.1 or less were assessed as straight.

**Curve walking and PCs 2, 4, and 5**

Flies with very low or very high mean values in the directions of PCs 2, 4, and 5 were analyzed with respect to the curvature of their walking trajectories and corresponding leg tip trajectories. Figure S2 shows exemplary walking trajectories of these flies with low and high means, together with the respective leg tip trajectories, which were color-coded according to the same code (Fig. S2D) as the walking trajectories. This color code differentiates between left and right turns, with blueish colors for right and yellowish colors for left turns. If remaining curve walking behavior is the cause of the differences between the extreme flies of any PC, we should see a clear tendency of yellowish leg-tip trajectories being on one side of the continuum and blueish ones on the other side. Instead, we see mostly equally distributed colors of leg-trajectories, while sometimes even fully yellow and blue ones overlap each other (e.g. Fig. S2Bii, right hind leg). PC 2 in Figure S2A features fly 1 (Aiii) and fly 3 (Aii) which are also shown in Figure 2 Bi and Biii, Figure 5, and Figure 8. Here we see how PC 2 describes the difference in posture width between both flies. Figure S3B shows low and high scoring examples along the dimension of PC 4, which gives an impression of potential curve walking behavior from the arrow depiction in Figure 3E, mainly because of the side shift in both front legs. Leg-tip trajectories of the extreme flies along the dimension of PC 4 shown in panels Bii and Biii in Figure S2 demonstrate that the individual posture preference is much more clearly correlated with PC 4 scores than the curvature of the walking trajectory. Hence, PC 4 describes first and foremost idiosyncrasies in walking behavior and is not directly correlated with remaining aspects of curve walking. Extreme cases of PC 5 in Figure S2C again show no correlation with trajectory curvature, but illustrate how PC 5 describes symmetric differences in posture which are mostly perpendicular to those described by PC 2. Panel D provides a legend for the color code of curvature values relative to the flies' body length.

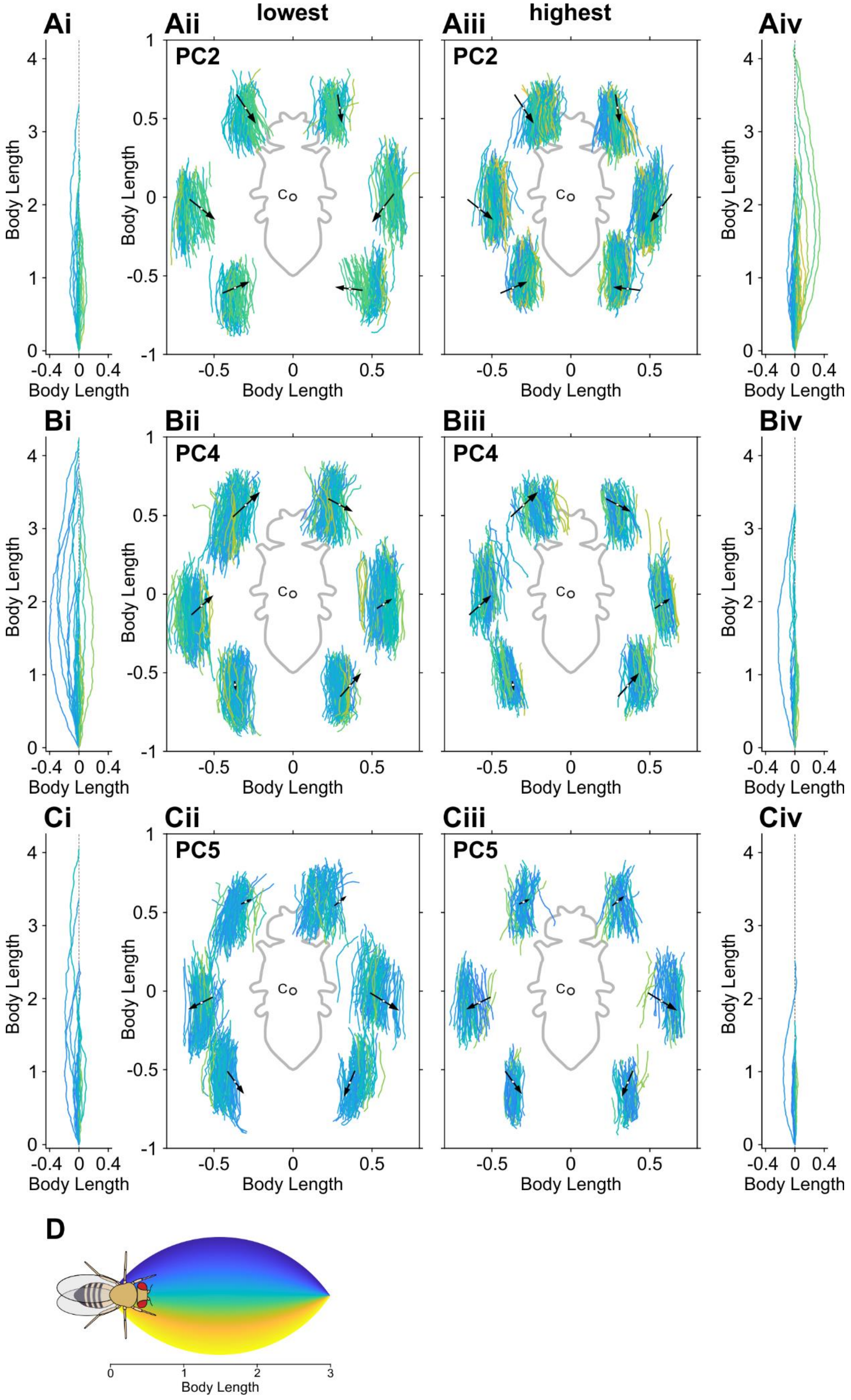


Journal of Experimental Biology • Supplementary information

**Fig. S2.** Walking trajectories and leg-tip stance trajectories of exemplary flies with low (i and ii) and high (iii and iv) scores for PCs 2, 4, and 5. (A) PC 2. (B) PC 4. (C) PC 5. Leg tip trajectories as well as trajectories are color-coded according to the curvature of the trajectory of their associated trial. (D) Color code for curvature of walking trajectories. Yellow hues indicate trajectories that are curved to the right, blue hues indicate trajectories that are curved to the left. (A) PC 2 mainly scales the overall posture width, with the fly scoring high (Aii) having a more sprawled posture than the one that scores low (Aii). (B) PC 4 most strongly affects the front legs (Bii and Biii, for high and low scores in PC 4) and due to its asymmetric nature suggests a curve walking context most directly. However, the associated trajectories (Bi and Biv, respectively) do not show a clear curve walking phenotype. (C) PC 5 mainly affects middle and hind legs. The curve walking implications for a fly that scores high (Cii) or low (Ciii) are very weak when the respective trajectories are evaluated (Ci and Civ, respectively). (D) Color code for curvature of walking trajectories. Yellow hues indicate trajectories that are curved to the right (positive values), blue hues indicate trajectories that are curved to the left (negative).

## Curvature vs PC scores

To more systematically test for correlations between slightly curved walking trajectories and certain PC scores we calculated the curvature of all recorded straight walking bouts in the data set and color coded it as in Figure S3D. We then calculated the fraction of variability described by all PCs for every individual walking bout and plotted the results in Figure S3. We also plotted regression lines, their slopes (m) and their $r^2$-values. Figure S3A shows a weak correlation for PC 2, as it explains only 1.9 % of the variance. The results for PC 4 in panel B show that it explains about 7.1 % of the curvature variance. This correlation is substantially weaker than the influence of individual preferences in average leg tip positions as demonstrated in Figure S2B. PC 5 in panel C does not show any clear correlation with only 0.2 % explained variance. The two PCs we identified as mostly related to aspects of interleg coordination (PCs 1 and 3, panels D and E) have similarly low coefficients of determination with regard to curvature and describe less than 1% and 1.8% of variance due to curvature, respectively.

Journal of Experimental Biology • Supplementary information

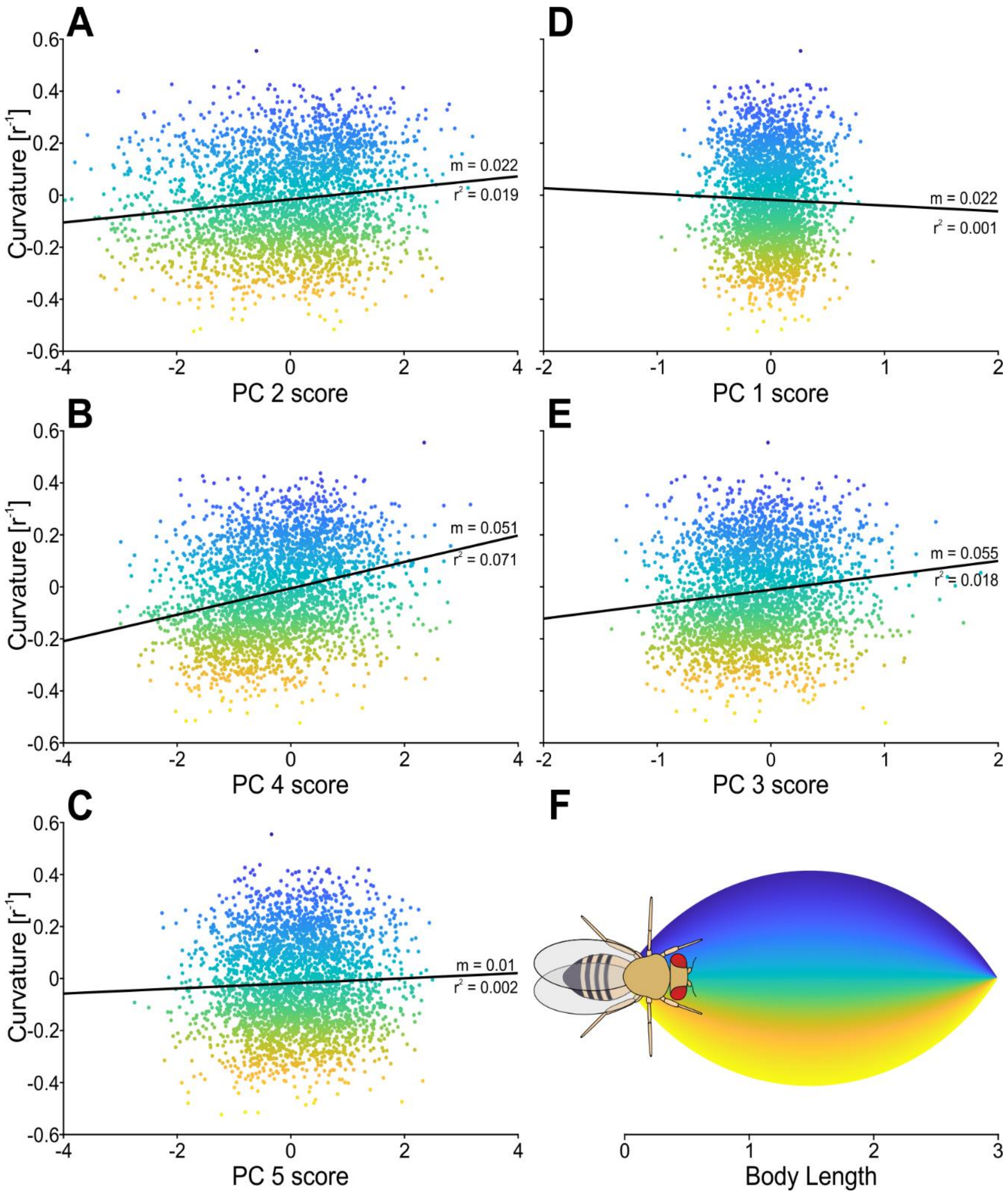


**Fig. S3.** Curvature of individual walking trajectories plotted against their respective scores in all PCs. (A to C) PCs 2, 4, and 5, respectively. (D and E) PCs 1 and 3. Black: regression line with slope (m) and coefficient of determination ($r^2$). (F) Color code for curvature of walking trajectories. Yellow hues indicate trajectories that are curved to the right (positive values), blue hues indicate trajectories that are curved to the left (negative). Coefficients of determination are generally very low, indicating no strong correlation between the curvature of walking trajectories and the described variance of the respective PC.

**Effect of walking speed on PC contributions**

Steps between 2 and 4 as well as between 8 and 10 BL $s^{-1}$ were transformed into the PC space resulting from the initial analysis that was based on a range of 5 to 7 BL $s^{-1}$ (see Fig. 6 in the main text). The results of this analysis results are shown in Figs. S4A and S4C, respectively. Fractions of described variability were calculated and plotted in the same way as in Figure 3A (for comparison, Fig. 3A was replicated here as Fig. S4B). Figure S4A shows the results for 2 to 4 BL $s^{-1}$, with a more than 10% smaller fraction of described variability for PC 1 and 1% smaller for PC3, while all other PCs described more variance compared to 5 to 7 BL $s^{-1}$. Panel C shows a continuation of the trend from A to B with even higher fractions of variability being described by PCs 1 and 3 while the described variability decreases for all other PCs. This result is a hint for an overall decrease of inter-individual variability with increasing walking speed, since relatively more variability is described by fewer PCs, particularly by PCs 1 and 3, which we identified as the ones that describe more general and invariant aspects of walking that all flies have.

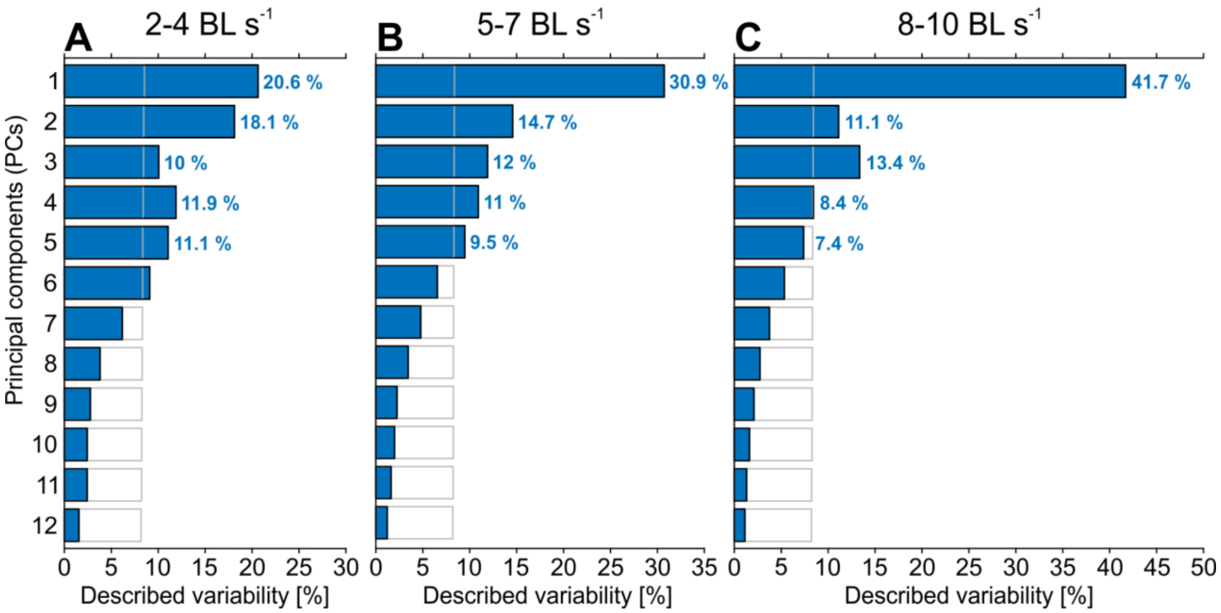


**Fig. S4.** Fractions of variability in leg tip kinematics described by each principal component for three different ranges of walking speed. Filled blue bars: Fractions of variability described by each principal component for the three subsets of data. Gray open bars: results for the same, but randomly permuted subsets of data (see results section). Percentages of PCs 1 to 5 are shown next to their respective bars. The minimum number of steps per fly was set to 30 for all conditions. (A) Subset of steps between 2 and 4 BL $s^{-1}$, n = 23 flies. (B) Original subset of steps between 5 and 7 BL $s^{-1}$ which was used for the PCA analysis (same data in Fig. 3A), n = 88 flies. (C) Subset of steps between 8 and 10 BL $s^{-1}$, n = 43 flies.

Journal of Experimental Biology • Supplementary information

## Walking speed-associated shifts in PC subspaces

Figure S5 shows the walking speed-associated changes of individual fly positions in the space spanned by PCs 2, 4, and 5 and PCs 1 and 3, respectively, (for absolute positions see Fig. 6 in the main text). Shifts are visualized as arrows. The left column (panels i) shows the shifts in position as they are located in the subspace. Flies with 30 or more steps in all three walking speed ranges are displayed with a single arrow with a bend at the position for 5 to 7 BL $s^{-1}$. All arrows point to the position of faster walking, while some start with the position at 5 to 7 BL $s^{-1}$ (if fewer than 30 steps were recorded between 2 and 4 BL $s^{-1}$) and others end with position at 5 to 7 BL $s^{-1}$ (if fewer than 30 steps were recorded between 8 and 10 BL $s^{-1}$). Flies which yielded the minimum of 30 or more steps for only one of the three conditions are not represented in this figure. The right column shows a centered version of the arrows shown on the left side for better comparison of their direction and length. Shifts are generally small and have no clear preference (also see panels ii for a centered version); this indicates that flies walked very similarly in the three respective walking speed ranges. Particularly the shifts in the space spanned by PCs 1 and 3 are almost non-existent. This supports the notion that these two PCs capture aspects of walking that are largely invariant and describe interleg coordination and overall movement of the legs. For more details see the Results section in the main text.

Journal of Experimental Biology • Supplementary information

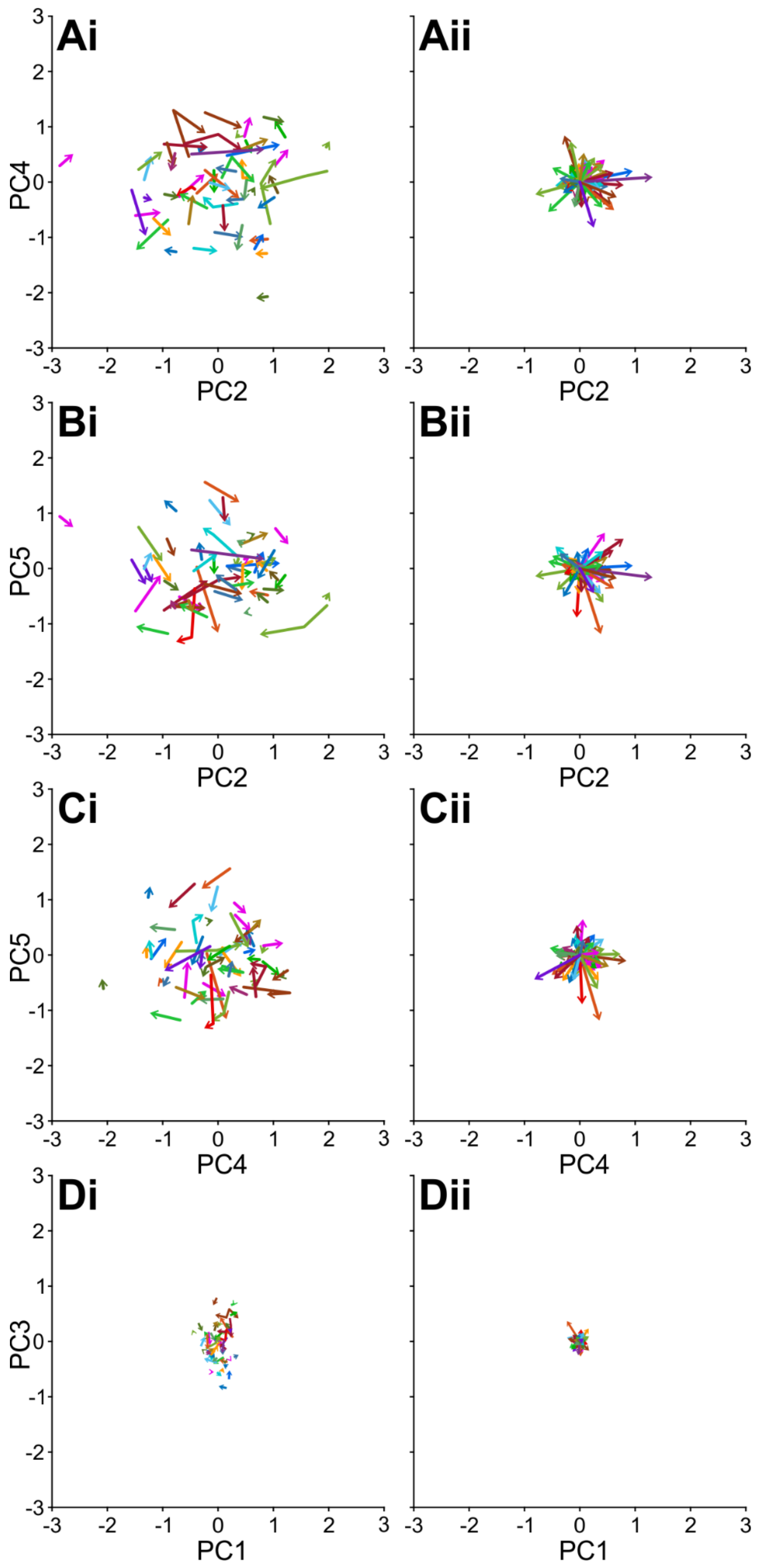


**Fig. S5.** Arrows showing the difference of average positions of individual flies for different walking speeds in the subspaces of PCs 1 to 5 shown in Fig. 6. The left column shows the absolute positions and their shifts as they are located in the subspace (also compare columns in Fig. 6). Flies with 30 or more steps in all three walking speed ranges are displayed with a single arrow with a bend at the position for 5 to 7 BL $s^{-1}$. All arrows point to the position of faster walking, while some start with the position at 5 - 7 BL $s^{-1}$ (if less than 30 steps were recorded between 2 and 4 BL $s^{-1}$) and

Journal of Experimental Biology • Supplementary information

others end with position at 5 - 7 BL $s^{-1}$ (if less than 30 steps were recorded between 8 and 10 BL $s^{-1}$). Flies which yielded the set minimum of 30 or more steps for only one of the three conditions are not represented in this figure. The right column shows a centered version of the arrows shown on the left side for better comparison of their direction and length. The color code is identical for both columns and matches the coloring in Figure 6. (A) Subspace of PCs 2 and 4. (B) Subspace of PCs 2 and 5. (C) Subspace of PCs 4 and 5. (D) Subspace of PCs 1 and 3.

**Recording time-associated shifts in PC subspaces**

Flies regularly spent 2 hours in the experimental setup and produced walking behavior throughout that time. Valid signatures of individuality (i.e. the positions of flies in the subspace of PCs 2, 4, and 5 we proposed as related to individual aspects of walking) should remain largely unchanged over the course of an experiment. To verify this, we analyzed how steps recorded during early and late phases of the session relate to each other in that subspace. For this purpose, we grouped trials of individual flies into an early and a late set. These two sets contained trials that were recorded at least 60 minutes apart from each other, but were produced at the same speed. Each set needed to contain at least 30 steps; 29 flies in total met these criterium. Analogously to the approach in the analysis of walking speed-dependent effects (see Fig. 6) we then transferred these subsets of data into the PC space and examined how different their average positions were in PC space (Fig. S6). Generally, the flies' average positions remained largely constant in the subspace spanned by PCs 2, 4, and 5 (Fig. S6, panels i to iii). While there were some shifts, many were small and in the order of the depicted SD ellipses (0.3 times the standard deviation) or smaller (compare vector lengths in panels Aiii to Ciii). At the same time, flies in the two conditions were still clearly distinguishable. Furthermore, there were no general directional trends in the shifts, making systematic changes in walking behavior due to exhaustion during a prolonged experiment, for instance, unlikely. Taken together, this supports the notion that PCs 2, 4, and 5 describe inter-individual differences in walking behavior that persist at least over the course of the experiments that were done here. The shifts i n the subspace spanned by PCs 1

Journal of Experimental Biology • Supplementary information

and 3 are even smaller; most shifts do not have an appreciable magnitude (Fig. S6Diii). This finding makes sense if we assume, that these two PCs describe variability in the walking behavior mostly related to coordination and speed and supports this notion even further.

There are a few singular exceptions. A particularly large shift was detected for fly 15 (purple color, Fig. S6Aiii to Ciii). Closer visual inspection of the underlying data revealed that, for unknown reasons, this particular fly clearly increased the spread of its hind legs towards the end of the recording session. While this individual is an exception of the rule and indeed did change its idiosyncratic way of walking, it became also evident that this produced a large signal in our analysis; however, combined with the fact that most flies did not produce these large signals this makes us more confident, that this approach can truly detect idiosyncrasies in walking behavior.

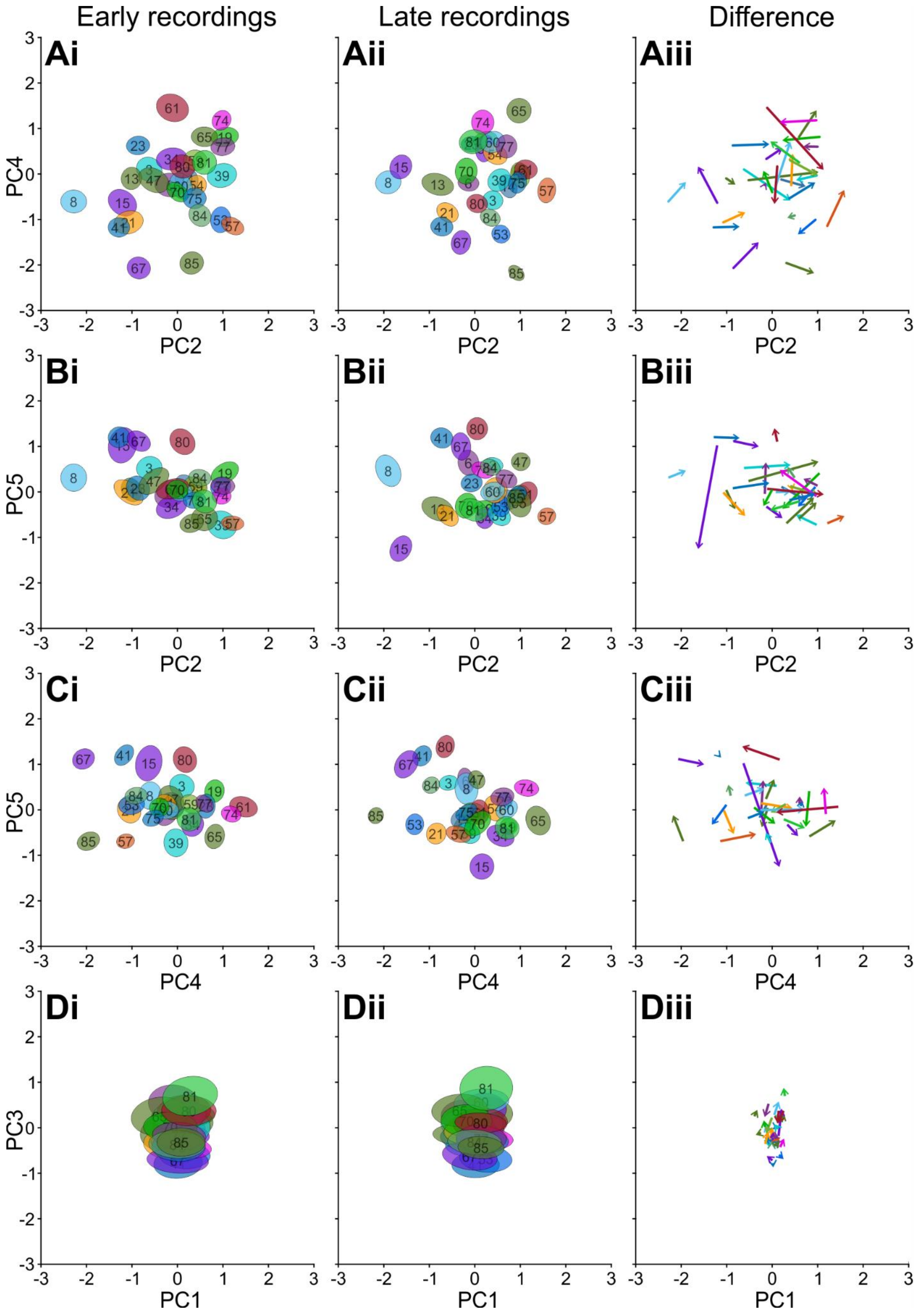


**Fig. S6.** Recording time-associated shifts in PC subspaces. First column: Average positions plus 0.3 times the standard deviations of flies for steps which were recorded early during the recording session. Second column: Average positions of flies for

Journal of Experimental Biology • Supplementary information

steps which were recorded at least 60 minutes after the last step of the early recordings group (first column). The minimum number of steps for early and late recording sets was 30 each. The resulting number of flies that produced enough steps in both sets was n = 29. Third column: Arrows showing the difference between average positions (center of ellipses) of early (first column) and late (second column) recordings of individual flies in the subspaces spanned by PCs 1 to 5. Arrows point from average positions in early recordings to the average position during later recordings. (A) Subspace of PCs 2 and 4. (B) Subspace of PCs 2 and 5. (C) Subspace of PCs 4 and 5. (D) Subspace of PCs 1 and 3.

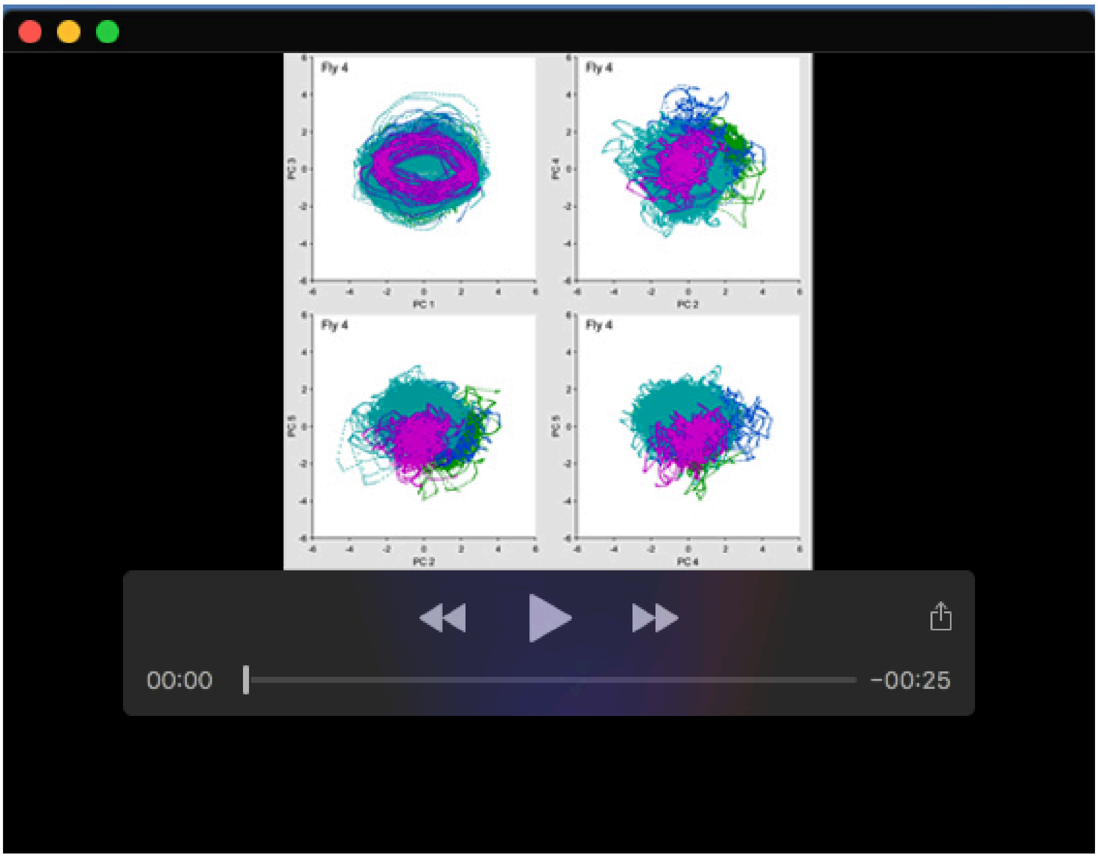


**Movie 1.** All data points (i.e. individual postures) of all flies in the analysis pool plotted, one after the other, on top of each other in 2D subspaces spanned by PCs 1 to 5. Arrangement as presented in Fig. 5. Individual colors correspond to individual flies (n = 88) but are not unique throughout the complete visualization. Note the relatively invariant elliptic shape of the data associated with individual flies in the subspace spanned by PC 1 and 3.

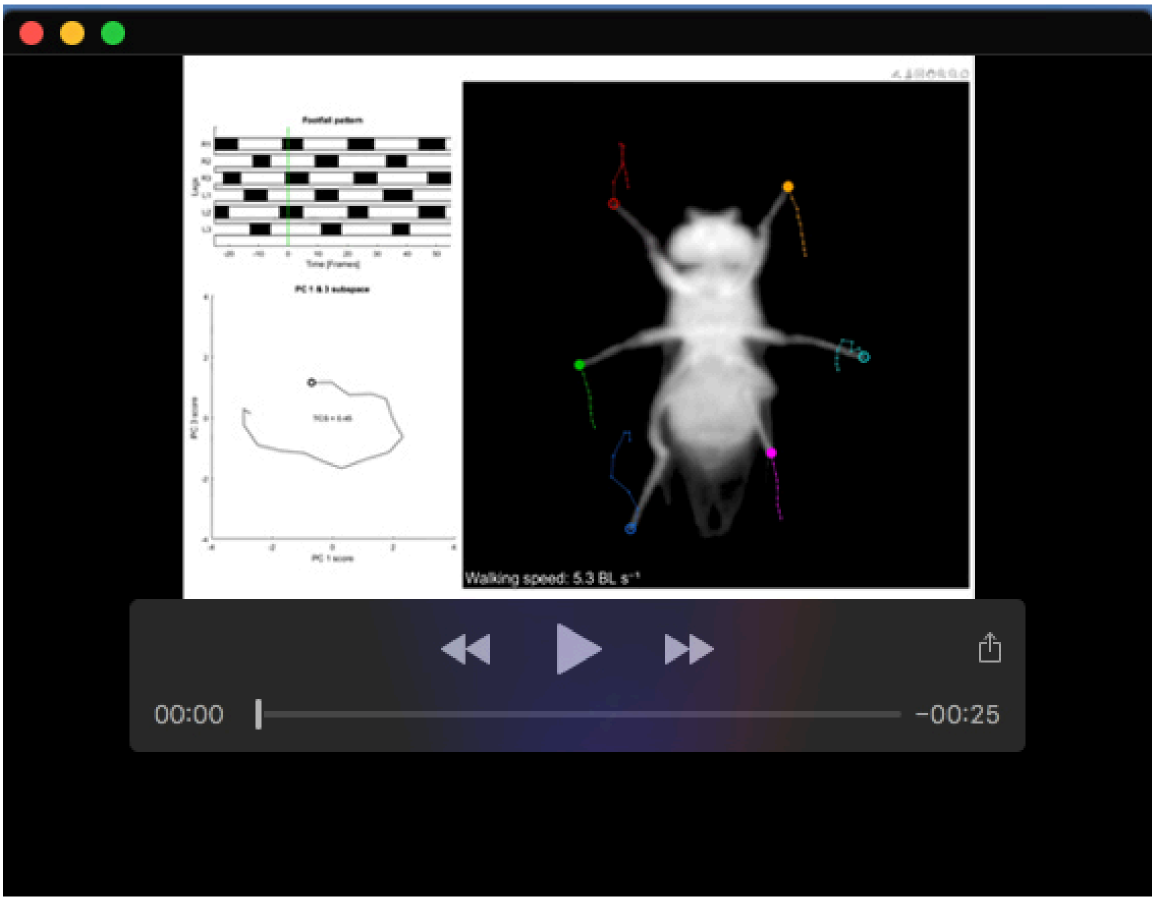


**Movie 2.** Example of a high-TCS walking bout with the respective footfall pattern and visualization of PCs 1 and 3. Footfall pattern: black indicates swing phase, white indicates stance phase. Video view: tracked leg tips (round markers) with parts of their prospective movements for clarity. Open markers indicate swing phase, filled markers indicate stance phase. Note that the walking speed is similar to that in Movie M3. Movie M2 shows relatively high TCS values, resulting in a relatively flat shape of the time courses of PCs 1 and 3, with major amplitudes of oscillation in the direction of PC 1. The resulting elliptic shape can be cut in half horizontally to differentiate between the swing phases of the left (lower half) and right (upper half) tripod group, respectively.

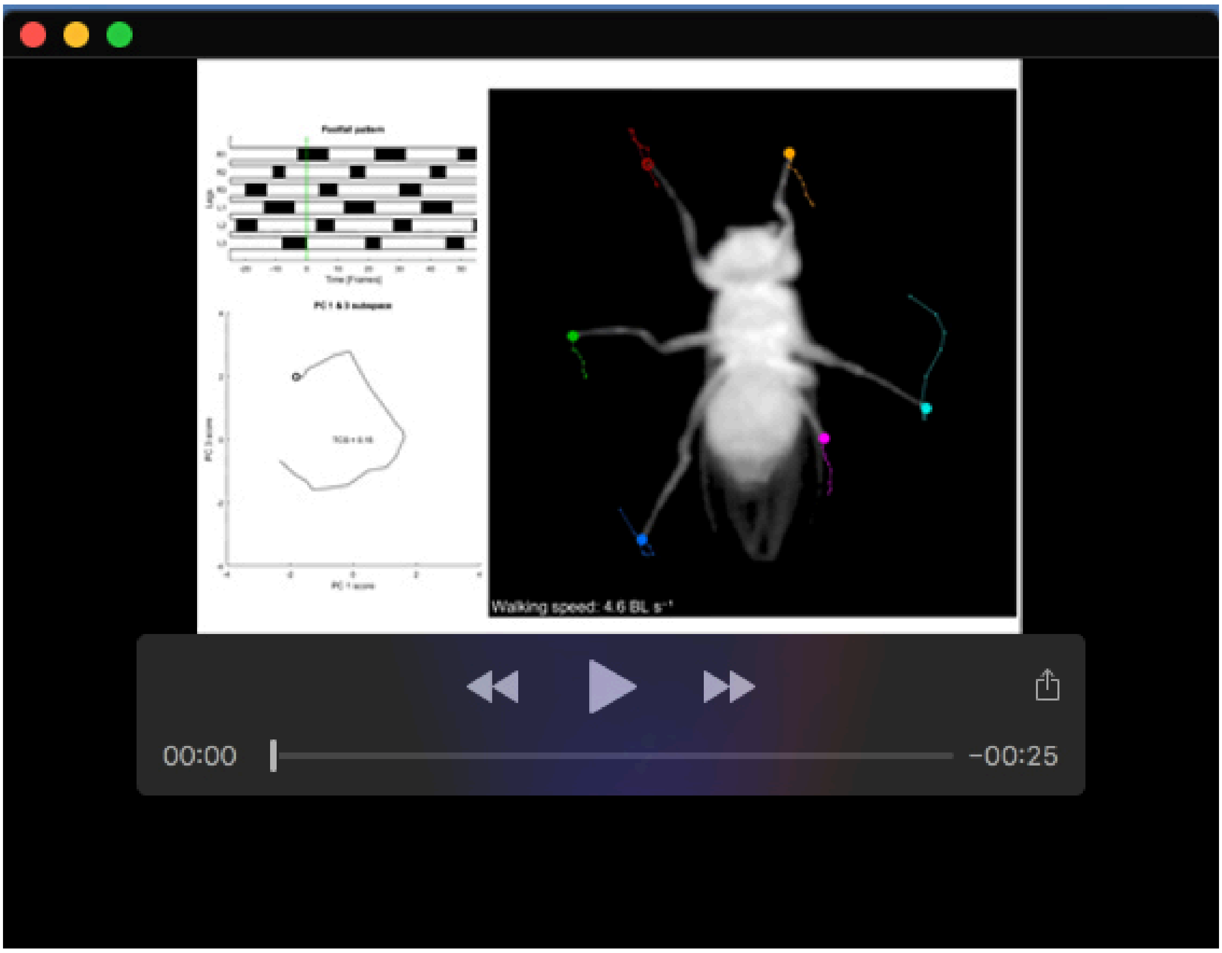


**Movie 3.** Example of a low-TCS walking bout with the respective footfall pattern and visualization of PCs 1 and 3. Footfall pattern: black indicates swing phase, white indicates stance phase. Video view: tracked leg tips (round markers) with parts of their prospective movements for clarity. Open markers indicate swing phase, filled markers indicate stance phase. Note that the walking speed is similar to that in Movie M2. Movie M3 shows relatively low TCS values, associated with increased amplitudes in direction of PC 3 and simultaneously decreased PC 1 activity. Unlike to the example in Movie M2, the differentiation of swing and stance activity of individual legs or tripod groups in the subspace spanned by PC 1 and 3 is not directly possible for low TCS examples, as the activity of individual legs does not align with the score time courses as during more tripod-like coordination.
